# A redescription of the Late Jurassic (Tithonian) turtle *Uluops uluops* and a new phylogenetic hypothesis of *Paracryptodira*

**DOI:** 10.1186/s13358-021-00234-y

**Published:** 2021-10-06

**Authors:** Yann Rollot, Serjoscha W. Evers, Walter G. Joyce

**Affiliations:** Department of Geosciences, University of Fribourg, Chemin de Musée 6, 1700 Fribourg, Switzerland

**Keywords:** *Testudinata*, *Baenidae*, *Compsemydidae*, *Helochelydridae*, *Pleurosternidae*, Phylogeny, Morphology, Neurobiology, Cranial circulation, Fossil record

## Abstract

We study the Late Jurassic (Tithonian) turtle *Uluops uluops* using micro-computed tomography scans to investigate the cranial anatomy of paracryptodires, and provide new insights into the evolution of the internal carotid artery and facial nerve systems, as well as the phylogenetic relationships of this group. We demonstrate the presence of a canalis caroticus lateralis in *Uluops uluops*, the only pleurosternid for which a palatine artery canal can be confidently identified. Our phylogenetic analysis retrieves *Uluops uluops* as the earliest branching pleurosternid, *Helochelydridae* within *Pleurosternidae*, and *Compsemydidae* including *Kallokibotion bajazidi* within *Baenidae*, which suggests at least two independent losses of the palatine artery within paracryptodires. We expect future studies will provide additional insights into the evolution of the circulation system of paracryptodires, as well as clarifying relationships along the turtle stem.

**Supplementary Information:**

The online version contains supplementary material available at 10.1186/s13358-021-00234-y.

## Introduction

*Uluops uluops* Carpenter and Bakker, 1990 is a poorly understood turtle known from a single skull from the Late Jurassic of North America. Carpenter and Bakker (1990) initially described *Uluops uluops* as a baenid paracryptodire closest to *Plesiobaena*, *Baena*, and *Eubaena* and diagnosed it as a new taxon based on a novel combination of primitive features found in non-baenid paracryptodires, e.g., a more rectangular, plate-like basitubera region, and derived features, such as the presence of a strongly curved maxillary cutting edge and a ventral muscle scar located along the basioccipital–basisphenoid suture. *Uluops uluops* was only recently included in a number of phylogenetic analyses focused on paracryptodires and was placed either in a basal polytomy at the base of *Baenoidea* (e.g., Lyson & Joyce, 2011; Perez-Garcia et al., 2015a, 2015b) or as the immediate sister of *Baenoidea* (e.g., Lyson et al., 2011; Joyce & Rollot, 2020). Although the differing hypotheses may be the result of true character conflict, the lack of a proper description of the only known specimen of *Uluops uluops* is equally problematic, as Carpenter and Bakker (1990) only provided a cursory description and idealized line drawings that lack sutures.

Paracryptodires were initially thought to have an extremely short canalis caroticus internus that splits into the canals for the cerebral and palatine arteries (respectively, the canalis caroticus basisphenoidalis and canalis caroticus lateralis sensu Rollot et al., 2021) upon entry to the skull midway along the parabasisphenoid–pterygoid suture (Gaffney, 1975). The palatine artery was thought to be reduced in size compared to the stapedial artery (Gaffney, 1972a, 1975). Using micro-computed tomography (µCT), Rollot et al. (2018) showed that the canal initially identified as the canalis caroticus lateralis in the Late Cretaceous baenid *Eubaena cephalica* (Gaffney, 1982) is actually the canalis nervus vidianus. This taxon, therefore, lacks a palatine artery. Using µCT scans once again, Evers et al. (2020) came to the same conclusion for the pleurosternid *Pleurosternon bullockii* Owen, 1842, which also had initially been reported as possessing a canalis caroticus lateralis (Evans & Kemp, 1975; Sterli et al., 2010). These observations coincide with those made for the putative early baenid *Arundelemys dardeni* Lipka et al., 2006, which was described as lacking the lateral canal based on µCT scans (Lipka et al., 2006). Although the presence of the canalis caroticus lateralis cannot be ruled out for taxa that have not been observed using µCT scans, such as *Dorsetochelys typocardium* (Anquetin & André, 2020) and *Glyptops ornatus* (Gaffney, 1979a), the limited data available to date suggest that the loss of a palatine artery may be a synapomorphy of *Paracryptodira*.

The phylogenetic relationships of the Cretaceous taxa *Kallokibotion bajazidi* Nopcsa, 1923 and *Helochelydridae* relative to *Paracryptodira* is under debate. In his original circumscription of *Paracryptodira*, Gaffney (1975) assigned *Kallokibotion bajazidi* to *Paracryptodira* based on the purported position of the foramen posterius canalis carotici interni located halfway along the pterygoid–parabasisphenoid suture, but later, manually produced trees suggesting that *Kallokibotion bajazidi* is sister to a paraphyletic *Baenoidea* and crown *Cryptodira* (Gaffney & Meylan, 1988, 1992). Global phylogenetic analyses of turtles have since provided varying results regarding the placement of *K. bajazidi*. Among others, Gaffney (1996) corroborated previous results of Gaffney and Meylan (1988, 1992), Hirayama et al. (2000) found *K. bajazidi* as sister taxon to the helochelydrid *Naomichelys speciosa* (their *Tretosternon*) outside the clade formed by *Baenoidea* and crown *Cryptodira*. Gaffney et al. (2007) found *K. bajazidi* as the sister taxon to monophyletic *Baenoidea* at the base of *Pan-Cryptodira*. Joyce (2007), Anquetin (2012), and Perez-Garcia and Codrea (2018) retrieved *K. bajazidi* as the immediate sister of crown Testudines, while Joyce et al. (2016) found *K. bajazidi* as the sister of the clade formed by *Baenoidea* and crown Testudines. *K. bajazidi* has occasionally also been found as a meiolaniform in global analyses that include both paracryptodires and meiolaniforms (Sterli et al., 2013), or at least in close proximity to meiolaniforms in a more stem-ward position than paracryptodires (e.g., Evers & Benson, 2019; see also review of Joyce, 2017). Despite the commonly hypothesized proximity of *K. bajazidi* to paracryptodires, phylogenetic analyses focused on the group never included this taxon (e.g., Lyson et al., 2011; Perez-Garcia et al., 2015a, 2015b; Joyce & Rollot, 2020). A recent review of paracryptodires reveals that numerous early forms can be united into the clade *Compsemydidae* by the retraction of the nuchal from the margin of the shell (Joyce & Rollot, 2020). This highly unusual characteristic, interestingly, occurs in *K. bajazidi* as well (Nopcsa, 1923; Perez-Garcia & Codrea, 2018), but has not been utilized in phylogenetic analyses.

Helochelydrids are relatively common fossils in the Cretaceous of Europe and North America, but their anatomy was only documented relatively recently (Joyce et al., 2011, 2014; Pérez-García et al. 2020). The few global phylogenetic analyses that include helochelydrids either place them as sister to *Baenoidea* just outside crown *Cryptodira* (Hirayama et al., 2000), as a distinct clade at the base of Perichelydia (Joyce et al., 2014), within *Meiolaniformes* at the base of Perichelydia (Anquetin, 2012), or in a paraphyletic grade with *K. bajazidi* and *Paracryptodira* (Joyce et al., 2016). Evers et al. (2020) recently highlighted numerous similarities of helochelydrids with the Late Jurassic pleurosternid *Pleurosternon bullockii* but these, too, have not been utilized in global phylogenetic analyses.

We here provide the first complete description of the holotype of *Uluops uluops* based on µCT scans, with a special focus on the carotid and facial nerve canal system. As this contribution is part of a larger project that aims to re-investigate paracryptodiran monophyly and relationships in a global context, and as similarities have recently been noted among baenoids with helochelydrids and *K. bajazidi*, we explicitly compare *U. uluops* to selected paracryptodiran and helochelydrid taxa and provide an expanded phylogenetic analysis of paracryptodire relationships.

### Geological setting

UCM (University of Colorado Museum of Natural History, Boulder, CO, USA) 53971 was collected at the Main Breakfast Bench Quarry at Como Bluff, Albany County, Wyoming, U.S.A (Carpenter & Bakker, 1990). The locality is located in the Brushy Basin Member of the Upper Jurassic (Tithonian) Morrison Formation, 17 m below the Dakota Sandstone (Turner & Peterson, 1999). The specimen was preserved in the Breakfast Bench Beds horizon, a thick, multi-storied trough crossbedded sandstone that also produced specimens of the dryolestid *Paurodon valens*, the multituberculate *Zofiabaatar pulcher*, and the neornithischian *Nanosaurus agilis* (Carpenter & Bakker, 1990).

## Materials and methods

UCM 53971 was subjected to high-resolution X-ray micro-computed tomography using a North Star Imaging scanner at the University of Texas High-Resolution X-ray Computed Tomography Facility, Austin, Texas, U.S.A. The custom-built scanner employs a gantry configuration based on the North Star Imaging X500 scanner and the detector is a 2048 × 2048 Perkin-Elmer flat-panel. Scanning was performed with 3000 projections over 360°, a voltage of 160 kV, a current of 200µA, and the use of an aluminum filter. 1896 coronal slices were obtained with a voxel size of 28.1 µm. 3D models were generated using the software Amira 2019.2 (https://www.fei.com/software) and reconstructions were obtained through interpolated slice-by-slice segmentation. The 3D models were exported as .ply-files and the software Blender 2.79b (https://www.blender.org) was used to create the images used in the figures. The original set of coronal slices were deposited at UCM and will be made available to qualified researchers. The 3D models generated as part of this study are available at MorphoBank (http://morphobank.org/permalink/?P3919).

The phylogenetic relationships of *Uluops uluops* were investigated by modifying the third paracryptodiran matrix employed by Joyce and Rollot (2020), which is based on Lyson and Joyce (2011). As our matrix only samples paracryptodires (and two basal testudinatans), our analyses are only suited to address paracryptodiran in-group relationships and provide no formal test of alternative positions for potential paracryptodiran taxa (such as *Kallokibotion bajazidi* and helochelydrids) with regard to their global position outside or inside of *Paracryptodira*. The matrix was expanded to include *Kallokibotion bajazidi* from the Maastrichtian of Romania, as described by Gaffney and Meylan (1992), Perez-Garcia and Codrea (2018), and Martín-Jiménez et al. (2021); the helochelydrid *Aragochersis lignitesta* Perez-Garcia et al., 2020 from the lower Albian of Spain, as described by Perez-Garcia et al. (2020); the helochelydrid *Helochelydra nopcsai* Lapparent de Broin and Murelaga, 1999 from Barremian of England, as described by Joyce et al. (2011) and personal observations of the type material housed at the Natural History Museum, London UK; and the helochelydrid *Naomichelys speciosa* from the Aptian–Albian of Texas, U.S.A., as described by Joyce et al. (2014). 11 new characters were added to the analysis (characters 97, 98, 99, 100, 101, 102, 103, 104, 105, 106, and 107 in our matrix), 6 were modified (16, 19, 27, 28, 80, and 88 in our matrix), and 7 were deleted (characters 24, 31, 38, 69, 75, 84, and 98 from the original matrix of Joyce & Rollot, 2020; see Additional file 2 for justifications). The analyses were subjected to a traditional parsimony analysis using TNT (Goloboff et al., 2008b). 21 characters that form morphoclines were run ordered (characters 6, 14, 16, 18, 27, 28, 31, 34, 39, 40, 41, 46, 48, 60, 63, 80, 88, 95, 97, 98, and 101). The initial analysis was carried out under equal weight and, following the recommendations of Goloboff et al., (2008a, 2018), implied weighting factors of *K* = 3, *K* = 6, *K* = 9, and *K* = 12 were implemented for a second set of analyses. 1000 random addition sequences were followed by a round of tree bisection reconnection. Trees suboptimal by 10 steps and with a relative fit difference of 0.1 were retained as part of the first search. A tree collapsing rule was implemented with a minimum length of 0. *Proganochelys quenstedti* was selected as the outgroup.

### Nomenclature

We use phylogenetic nomenclature as recently codified by the PhyloCode (Cantino and de Queiroz, 2020). All clade names used herein are highlighted as such through the use of italics.

Our anatomical nomenclature follows that of Gaffney (1972b) with modifications in regard to the terminology of the carotid and facial nerve system as recently summarized by Rollot et al. (2021). Following the rationale outlined by Rollot et al. (2021), however, we introduce two additional terms, which are modifications of previous terms introduced by Rabi et al. (2013b).

Foramen posterius canalis carotici basisphenoidalis—the posterior foramen to the canalis caroticus basisphenoidalis (foramen posterius canalis carotici cerebralis of Rabi et al., 2013b), which serves as an entry for the cerebral artery into the skull. The foramen posterius canalis carotici basisphenoidalis is typically located in the lateral margin of the parabasisphenoid and concealed when the carotid split is not exposed. The foramen posterius canalis carotici basisphenoidalis is exposed on the ventral side of the skull when the branching off of the internal carotid artery into its primary branches is not ventrally covered by bone. We favor the term foramen posterius canalis carotici basisphenoidalis over foramen posterius canalis carotici cerebralis, as the artery traversing the canal may supply blood to more tissues than just the brain and because it restores a connection with a significant body of literature (Rollot et al., 2021).

Foramen posterius canalis carotici lateralis—the posterior foramen to the canalis caroticus lateralis (foramen posterius canalis caroticus palatinum of Rabi et al., 2013b), which serves as an entry for the palatine artery into the skull. The foramen posterius canalis carotici lateralis is located along the pterygoid–parabasisphenoid suture lateral to the foramen posterius canalis carotici basisphenoidalis, and typically exposed on the ventral side of the skull when the branching off of the internal carotid artery into its primary branches is not ventrally covered by bone. We favor the term foramen posterius canalis carotici lateralis over foramen posterius canalis carotici palatinum, as the canal may contain arteries other than palatine artery and because it restores a connection with a significant body of literature (Rollot et al., 2021).

### Systematic paleontology

*Testudinata* Klein, 1760 [Joyce et al., 2020].

*Paracryptodira* Gaffney, 1975 [Joyce et al., 2021].

*Pleurosternidae* Cope, 1868 [Joyce et al., 2021].

*Uluops uluops* Carpenter & Bakker, 1990

#### Type specimen

UCM 53971 (holotype), a cranium (Carpenter & Bakker, 1990: Fig. 4).

#### Type locality

Main Breakfast Bench Quarry, Albany County, Wyoming, U.S.A. (Carpenter & Bakker, 1990); Morrison Formation, Tithonian, Late Jurassic.

#### Referred material and range

None.

#### Emended diagnosis

*Uluops uluops* can be diagnosed as a pleurosternid by the presence of a jugal that does not extend deeply ventrally, a plate-like supraoccipital exposure on the skull roof between the parietals, an anteriorly convex nasal–frontal suture, anterior tubercula basioccipitale on the parabasisphenoid, and the exclusion of the exoccipitals from the articular surface of the occipital condyle. *Uluops uluops* is distinguished from all other pleurosternids by the following combination of features: a reduced lingual ridge anteriorly, a foramen palatinum posterius entirely formed by the palatine, a midline contact of the pterygoids of about 10–40% of their length, a length between orbit and cheek emargination equal to the diameter of the orbit, a maximum combined width of the parietals greater than their length, the presence of a canalis caroticus lateralis, a raised ridge on the dorsal surface of the paroccipital process, and anterior abducens nerve foramina that are entirely formed by the pterygoid.

### Description

#### Skull

**Fig. 1:**
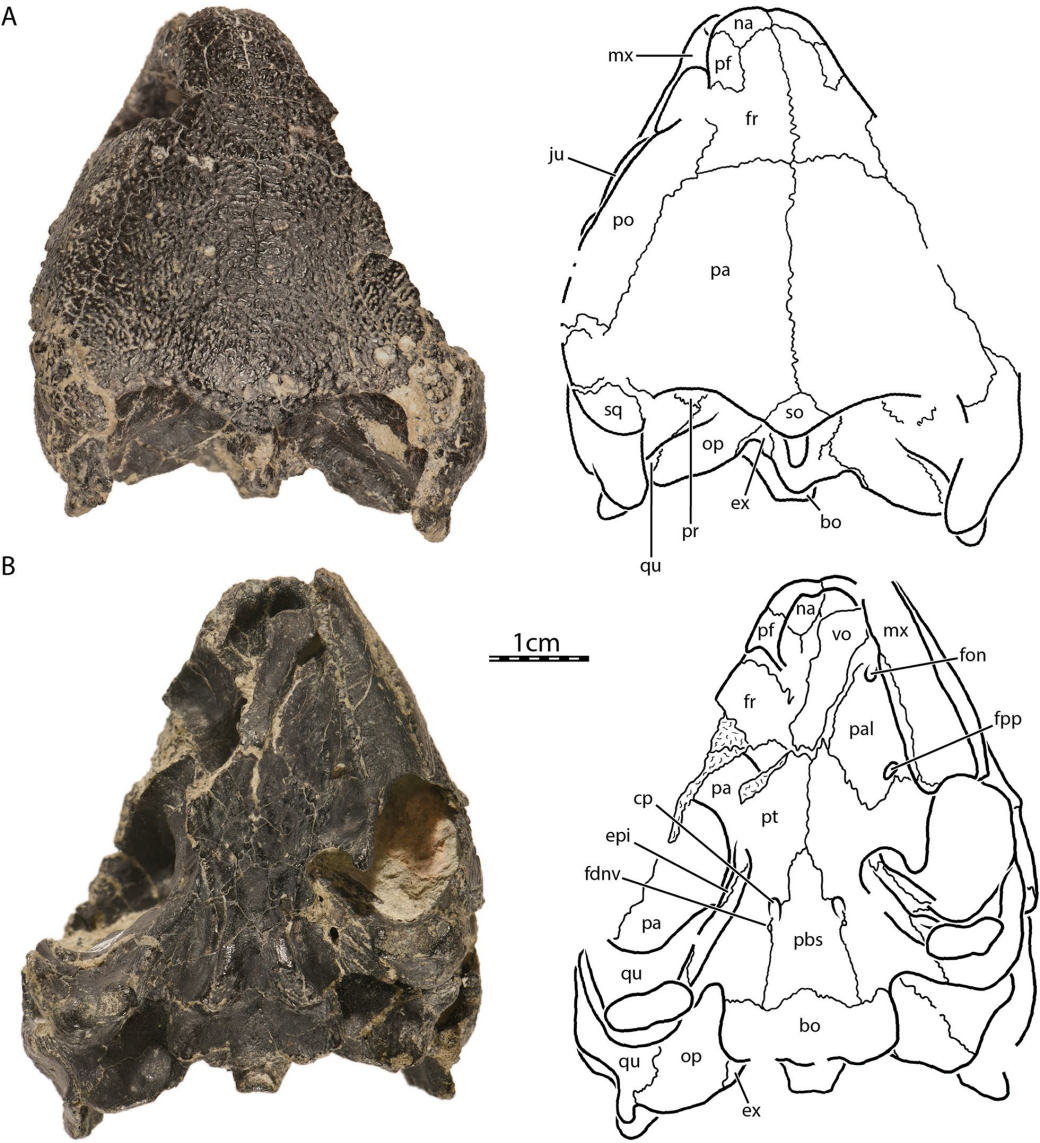
*Uluops uluops*, UCM 53971, Late Jurassic (Tithonian) of Wyoming, U.S.A. Photographs and illustrations of skull in **A** dorsal, and **B** ventral views. *bo* basioccipital, *cp* carotid pit, *ex* exoccipital, *fdnv* foramen distalis nervi vidiani, *fon* foramen orbito-nasale, *fpp* foramen palatinum posterius, *fr* frontal, *ju* jugal, *mx* maxilla, *na* nasal, *op* opisthotic, *pa* parietal, *pal* palatine, *pbs* parabasisphenoid, *pf* prefrontal, *po* postorbital, *pt* pterygoid, *qu* quadrate, *so* supraoccipital, *sq* squamosal, *vo* vomer

**Fig. 2:**
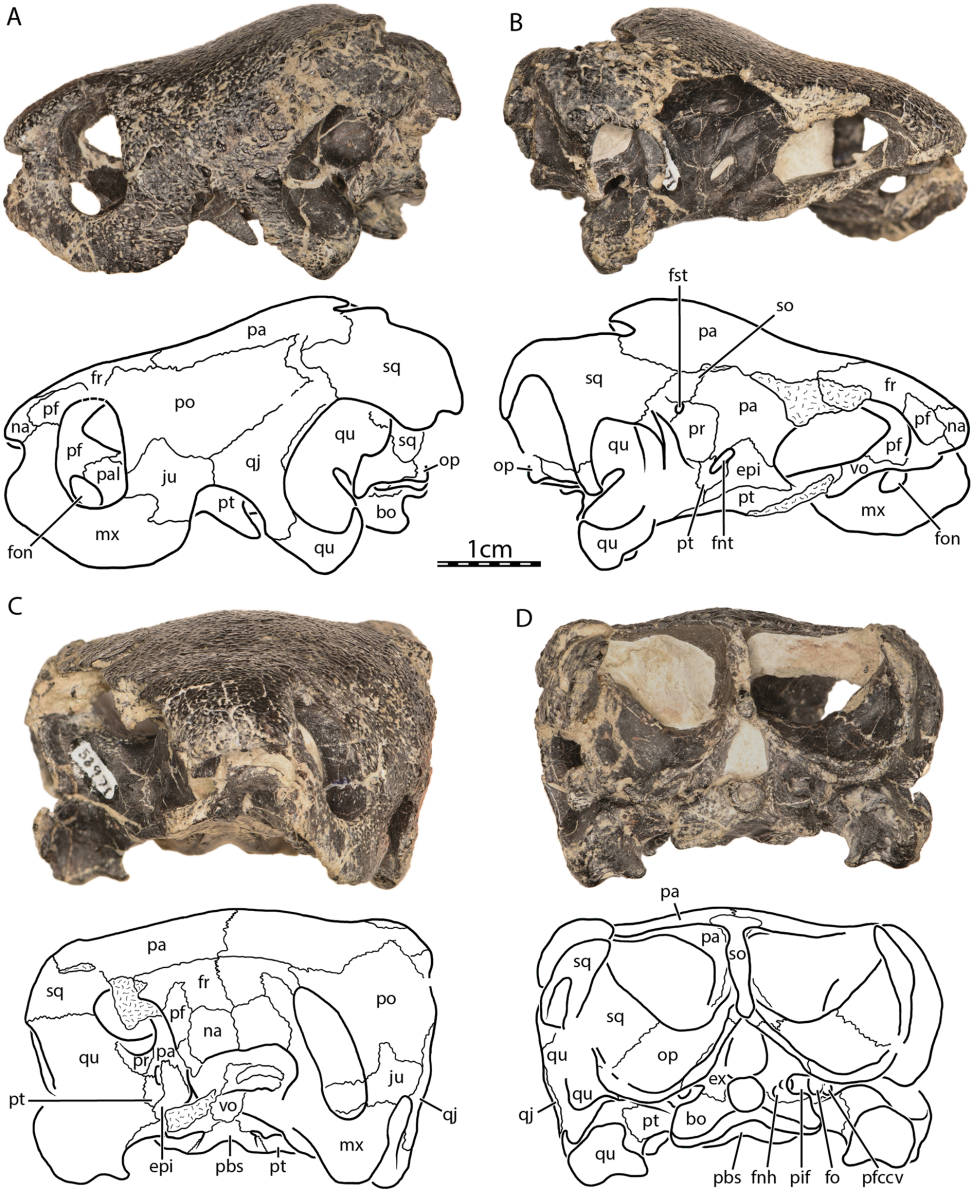
*Uluops uluops*, UCM 53971, Late Jurassic (Tithonian) of Wyoming, U.S.A. Photographs and illustrations of skull in **A** left lateral, **B** right lateral, **C** anterior, and **D** posterior views. *bo* basioccipital, *epi* epipterygoid, *ex* exoccipital, *fnh* foramen nervi hypoglossi, *fnt* foramen nervi trigemini, *fo* fenestra ovalis, *fon* foramen orbito-nasale, *fr* frontal, *fst* foramen stapedio-temporale, *ju* jugal, *mx* maxilla, *na* nasal, *op* opisthotic, *pa* parietal, *pal* palatine, *pbs* parabasisphenoid, *pf* prefrontal, *pfccv* posterior foramen for the canalis cavernosus, *pif* processus interfenestralis of the opisthotic, *po* postorbital, *pr* prootic, *pt* pterygoid, *qj* quadratojugal, *qu* quadrate, *so* supraoccipital, *sq* squamosal, *vo* vomer

**Fig. 3:**
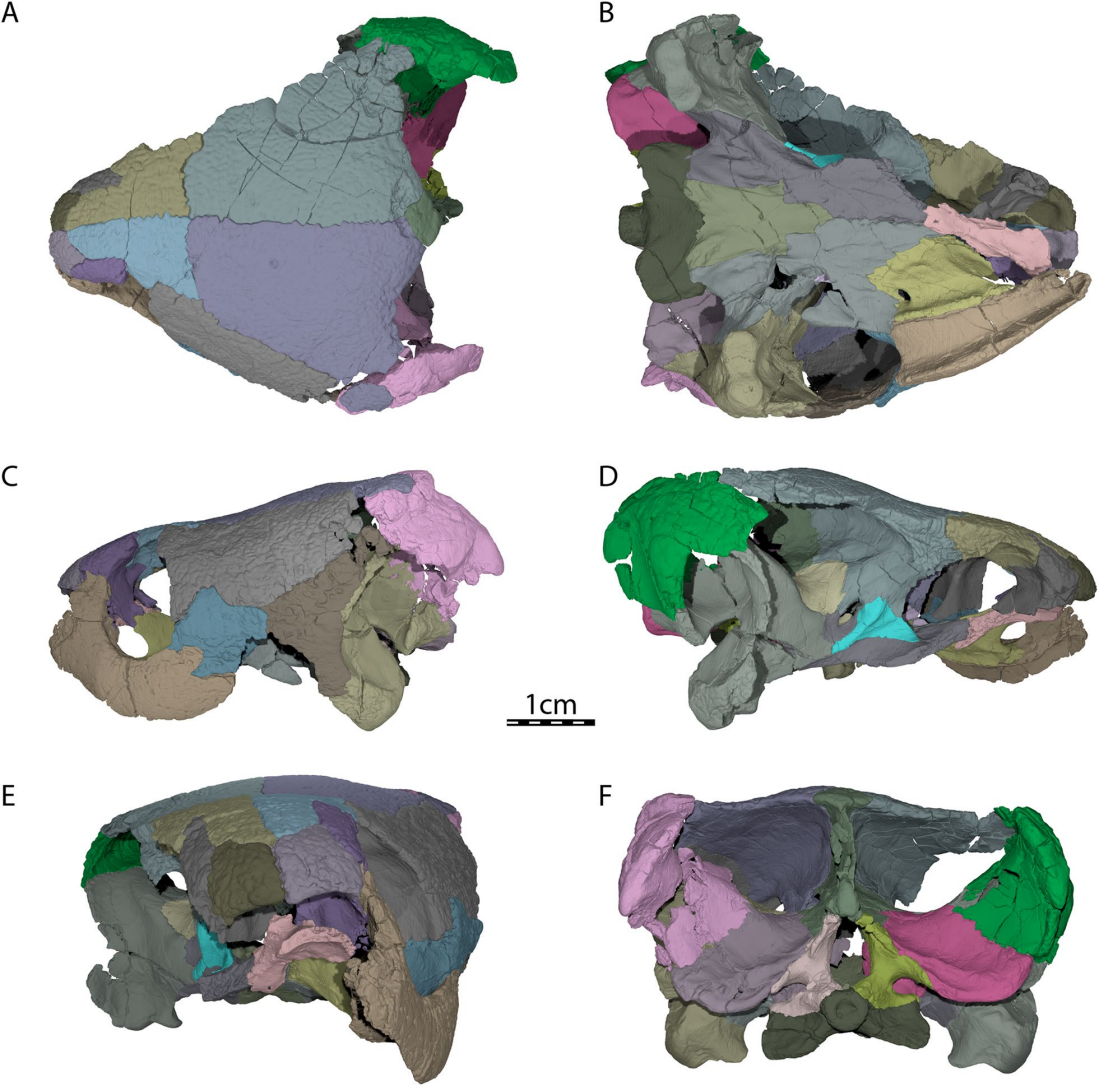
Three-dimensional renderings of UCM 53971. **A** Dorsal view, **B** ventral view, **C** left lateral view, **D** right lateral view, **E** anterior view, and **F** posterior view

The skull of UCM 53971 is well preserved, as most of the preserved bones remain in articulation and show only minor signs of crushing (Figs. 1, 2, 3). However, a substantial part of the right side of the skull is missing, in particular the right maxilla, palatine, postorbital, jugal, quadratojugal, and both premaxillae (Fig. 2B). The left otic capsule is heavily damaged externally and internally by a multitude of cracks. The vomer is slightly displaced towards the left, but its sutural contacts with the surrounding bones are preserved (Fig. 1B). The good preservation of at least one of each paired bone with the exception of the premaxillae allows for detailed observation of every bone and all internal structures.

The skull of UCM 53971 resembles that of baenodds by being relatively short, broad, and highly domed (Figs. 1A and 2A). The orbits are laterally oriented as in *Compsemys victa* (Lyson & Joyce, 2011). The skull is nearly as long as wide. The length reaches 44.4 mm from the foramen magnum to the anterior tip of the nasals while the width is 39.6 mm between the squamosals dorsal to the cavum tympani, which is the maximum width of the cranium. The lateral surfaces of the skull are vertically oriented (Fig. 2C), as was mentioned by Carpenter and Bakker (1990). The maxilla, jugal, quadratojugal, and quadrate jointly form a distinct cheek emargination that crosses an imaginary line between the lower margin of the orbit and the incisura columella auris (Fig. 2A). The cheek emargination is similarly developed in *Pleurosternon bullockii* (Evans & Kemp, 1975), but is reduced to absent in *Compsemys victa* (Lyson & Joyce, 2011), *Glyptops ornatus* (Gaffney, 1979a) and *Dorsetochelys typocardium* (Evans & Kemp, 1976). The upper temporal emargination is moderately concave and is mainly bordered by the squamosals and parietals, with the supraoccipital only forming the posteromedial margin. In dorsal view, the skull roof covers most of the otic capsule and the entire foramen stapedio-temporale (Fig. 1A). This arrangement resembles that of *Dorsetochelys typocardium* (Evans & Kemp, 1976), *Glyptops ornatus* (Gaffney, 1979a), and *Pleurosternon bullockii* (Evans & Kemp, 1975), but contrasts with *Compsemys victa* (Lyson & Joyce, 2011), which likely lacks an upper temporal emargination. The crista supraoccipitalis extends only slightly posteriorly to the foramen magnum (Fig. 1A). No cranial scute sulci are visible on the dorsal skull surface. The skull surface is marked by small and irregular tubercles (Fig. 1A), which resemble those of *Glyptops ornatus* (Gaffney, 1979a), *Pleurosternon moncayensis* (Perez-Garcia et al., 2021), and *Pleurosternon bullockii* (Evans & Kemp, 1975), although the latter exhibits a more crenulated like pattern towards the back of the skull roof, and differ from the weakly crenulated pattern of *Arundelemys dardeni* (Lipka et al., 2006) and *Compsemys victa* (Lyson & Joyce, 2011).

#### Nasal

The nasal is moderately large, approximately as long as wide, and overall similar to that of other non-baenodd paracryptodires (Figs. 1A and 2C). In dorsal view, the nasal contacts its counterpart medially for two-thirds of its anteroposterior length. Although the nasals slightly diverge with their posterior margin from the midline, they are not fully separated by the anterior processes of the frontals, as is the case in *Pleurosternon bullockii* (Evers et al., 2020). On the dorsal skull surface, the nasal contacts the frontal posteromedially, the prefrontal posterolaterally, and the maxilla posteroventrally (Fig. 1A). At its posterior end, the nasal forms a transverse facet that braces the frontal dorsally and ventrally for a short distance. The nasal forms the dorsal margin of the apertura narium externa and roofs the nasal cavity (Fig. 2C). The ventral exposure of the nasal within the nasal cavity is smaller than its external exposure on the dorsal skull roof, as the nasal is partially underlain by the prefrontal. Within the nasal cavity, the nasal contacts its counterpart anteromedially and forms a shallow median ridge that separates the nasal cavity into right and left nasal valves.

#### Prefrontal

The prefrontal contributes to the formation of the nasal cavity and the anterodorsal margin of the orbit (Figs. 2A and C). The dorsal plate of the prefrontal is exposed dorsally but does not contact its counterpart medially. The ventral exposure of the prefrontal within the nasal cavity is greater than its dorsal exposure, as the prefrontal broadly underlies a portion of the frontal. The dorsal plate contacts the maxilla anteroventrally, the nasal anteromedially, and the frontal posteromedially and posteriorly (Figs. 1A, 2A and C). Within the orbit, the suture between the prefrontal and frontal is strongly interdigitated, forming a W-shaped suture in ventral view. The thin, descending process of the prefrontal extends ventrally to form the anteromedial wall of the fossa orbitalis. The descending process of the prefrontal likely formed the anterodorsal margin of the large foramen orbito-nasale, but damage obscures this region. The descending process contacts the maxilla anterolaterally, the palatine ventrally, and the vomer ventromedially. Other non-baenodd paracryptodires exhibit similar contacts and contributions of the prefrontal to various cranial structures as UCM 53971.

#### Frontal

The frontal is a subtriangular bone that is about twice as long antero-posteriorly as it is wide mediolaterally at its widest part, which is located at its posterior contact with the parietal (Fig. 1A). The frontal forms an orbital process at its mid-length that inserts between the prefrontal anteriorly and the postorbital posteriorly, thereby preventing these bones from contacting each other (Figs. 1A and 2A). This process contributes to the dorsal margin of the orbit. *Compsemys victa* (Lyson & Joyce, 2011) differs from that condition as the prefrontal and postorbital contact each other along the dorsal margin of the orbit, preventing the frontal from contributing to the formation of the latter. Anterior to the orbital process of UCM 53971, the anterior processes of the frontal have parallel margins and fully separate the prefrontals from one another (Figs. 1A and 2C). At the anterior end, the frontal margin tapers medially along its contact with the nasal (Fig. 1A). This taper has a similar extent in *Dorsetochelys typocardium* (Evans & Kemp, 1976) and *Glyptops ornatus* (Gaffney, 1979a), is more extensively developed in *Pleurosternon bullockii* (Evans & Kemp, 1975), but is lacking in *Compsemys victa* (Lyson & Joyce, 2011). The anterior process of the frontal tapers more gradually within the roof of the fossa orbitalis of UCM 53971. Each frontal has a shallow, but well-developed ventral ridge, the crista cranii, which collectively form the sulcus olfactorius.

#### Parietal

The parietal is about twice as wide posteriorly as anteriorly and consists of a dorsal and a ventral plate (Figs. 1A and 2B). The dorsal plate roofs the braincase and forms most of the weakly developed upper temporal emargination and the posterior limit of the skull roof. In dorsal view, the parietal fully conceals the otic capsule, including the foramen stapedio-temporale (Fig. 1A). A weakly developed upper temporal emargination is also found in *Dorsetochelys typocardium* (Evans & Kemp, 1976), *Glyptops ornatus* (Gaffney, 1979a), and *Pleurosternon bullockii* (Evans & Kemp, 1975). On the dorsal skull roof of UCM 53971, the parietal contacts its counterpart along the midline, the frontal anteriorly, the postorbital anterolaterally and laterally, the squamosal posterolaterally, and the supraoccipital posteromedially (Fig. 1A). The ventrally descending vertical plate of the parietal, the processus inferior parietalis, has a broad contact with the epipterygoid anterior to the foramen nervi trigemini, a short contact with the pterygoid posterior to the foramen nervi trigemini, a broad contact with the prootic within the upper temporal fossa, and a broad posterior contact with the supraoccipital within the upper temporal fossa (Fig. 2B). The processus inferior parietalis forms the anterior part of the lateral wall of the cavum cranii, the medial margin of the fossa temporalis, the posterior margin of the foramen interorbitale, and the dorsal and posterodorsal margins of the foramen nervi trigemini (Fig. 2B). The parietal forms a minor anterolateral process that partially underlies the postorbital and that forms a mediolateral ridge visible in the roof of the temporal fossa. This ridge forms the posterior limit of the sulcus palatino-pterygoideus, which is anteriorly bordered by the posterodorsal margin of the fossa orbitalis and which is developed similarly to that of *Pleurosternon bullockii* (Evers et al., 2020). Posterior to the foramen nervi trigemini, the processus inferior parietalis bears a posteroventrally oriented process that contacts the pterygoid along the posterior margin of the foramen nervi trigemini (Fig. 2B). This process prevents the prootic from participating in the formation of the foramen nervi trigemini, a condition that is also observed in *Pleurosternon bullockii* (Evers et al., 2020) and *Pleurosternon moncayensis* (Perez-Garcia et al., 2021), but not in *Compsemys victa* (Lyson & Joyce, 2011) and *Glyptops ornatus* (Gaffney, 1979a).

#### Postorbital

The postorbital is an elongate, plate-like bone that is about twice as long as broad and that contributes to the formation of the dorsolateral skull roof (Figs. 1A and 2A). The postorbital contacts the jugal anteroventrally along a sinusoid-shaped suture, the quadratojugal posteroventrally, the frontal anterodorsally, the parietal mediodorsally, and the squamosal posteriorly (Figs. 1A and 2A). The broad contact between the parietal and squamosal posterior to the postorbital prevents the latter from contributing to the formation of the upper temporal emargination (Fig. 1A). The postorbital forms the posterior margin of the orbit and the posterior wall of the fossa orbitalis (Fig. 2A). Within the fossa orbitalis, the postorbital has a flat, slightly oblique contact with the jugal. The medial process of the jugal prevents any contact of the postorbital with the maxilla, pterygoid, or palatine. Together with the jugal, the postorbital forms a robust posterior wall of the fossa orbitalis, reminiscent of the condition seen in pleurodires and trionychids, although the postorbital reaches the pterygoid and palatine bones in these taxa (Gaffney, 1979b). This extended posterior wall of the fossa orbitalis is also present in *Compsemys victa* (Lyson & Joyce, 2011), *Glyptops ornatus* (Gaffney, 1979a), and *Pleurosternon bullockii* (Evers et al., 2020).

#### Jugal

The jugal forms the posteroventral portion of the fossa orbitalis and makes a small contribution to the posteroventral margin of the orbit (Figs. 2A and C). This contribution to the orbit is also present in *Compsemys victa* (Lyson & Joyce, 2011), but absent in *Dorsetochelys typocardium* (Evers et al., 2020), *Glyptops ornatus* (Gaffney, 1979a), and *Pleurosternon bullockii* (Evers et al., 2020). Within the fossa orbitalis of UCM 53971, the jugal contacts the maxilla anteriorly and broadly rests on the posterior part of this bone along a horizontal suture (Fig. 2C). The medial process of the jugal contacts the palatine medially and the pterygoid posteromedially. On the dorsolateral skull surface, the jugal otherwise contacts the postorbital dorsally and the quadratojugal posteriorly (Fig. 2A). Lastly, the jugal forms the anterodorsal margin of the lower temporal emargination, which only rises just above the lower margin of the orbit, but appears accentuated by the brevity of the skull and the depth of the maxilla (Fig. 2A).

#### Quadratojugal

The quadratojugal is a thin, triradiate element. The posterior rim of the quadratojugal is concave and forms the anteroventral margin of the cavum tympani (Fig. 2A). The quadratojugal also forms the posterior half of the cheek emargination. The anterior process of the quadratojugal contacts the jugal anteriorly. The quadratojugal otherwise contacts the postorbital anterodorsally, the squamosal posterodorsally, and the quadrate posteriorly and posteroventrally (Fig. 2A). The quadratojugal of UCM 53971 is overall similar to that of *Pleurosternon bullockii* (Evans & Kemp, 1975) and to *Dorsetochelys typocardium* (Evans & Kemp, 1976), which, however, has a taller anterior process.

#### Squamosal

The squamosal is a complex element that lies at the posterodorsolateral margin of the skull (Figs. 1A and 2). It forms the antrum postoticum, the posterodorsal margin of the cavum tympani, and the lateral margin of the upper temporal emargination. On the skull roof, the squamosal contacts the postorbital anteriorly, likely the quadratojugal anteroventrally, the quadrate ventrally, and the parietal anteromedially (Figs. 1A and 2A). Within the temporal fossa, the squamosal contacts the quadrate anteromedially and the opisthotic posteromedially (Figs. 1A and 2D). The squamosal forms a distinct, posteromedially directed curved flange that represents the posterior-most aspect of the skull (Fig. 1A). A broad concavity is developed along the lateral margin of this flange, which is likely the attachment site of the m. depressor mandibulae (Werneburg, 2011).

#### Premaxilla

The premaxillae are not preserved.

#### Maxilla

The maxilla forms the anterior and ventral margins of the orbit and the anterolateral aspects of the floor of the fossa orbitalis (Figs. 2A and C). In ventral view, the maxilla contacts the palatine medially along the margin of the triturating surface, the pterygoid posteromedially, and the jugal posterolaterally (Fig. 1B). Although the vomer is slightly displaced, a contact between the maxilla and the vomer was likely present anterior to the apertura narium interna. The ascending process of the maxilla forms the lateral margin of the apertura narium externa and contacts the nasal anterodorsally and the prefrontal posterodorsally (Figs. 2A and C). The foramen alveolare superius is formed as an unusually large opening located at the medial base of the ascending process, ventrally underneath the margin of the foramen orbito-nasale. On the lateral surface of the skull, the maxilla contributes to the formation of the anteroventral margin of the cheek emargination and contacts the jugal along a z-shaped suture (Fig. 2A). Within the fossa orbitalis, the maxilla forms the anterolateral margin of the foramen orbito-nasale, broadly contacts the palatine medially, and is posterodorsally covered by the jugal (Figs. 2A and C). The maxilla forms along with a minor contribution from the palatine a relatively narrow triturating surface that expands towards the posterior and that is bordered laterally by a high labial ridge (Fig. 1B). High labial ridges are also present in *Compsemys victa* (Lyson & Joyce, 2011) and *Dorsetochelys typocardium* (Evans & Kemp, 1976). The labial ridge and the triturating surface of UCM 53971 are strongly convex along the anteroposterior length of the maxilla. This curvature is intermediate in *Pleurosternon bullockii* (Evans & Kemp, 1975) and only weakly developed in *Compsemys victa* (Lyson & Joyce, 2011) and *Glyptops ornatus* (Gaffney, 1979a). Carpenter and Bakker (1990) noted that the triturating surfaces of UCM 53971 are much wider than in *Glyptops ornatus* and *Pleurosternon bullockii*. We agree with their comparison with *Glyptops ornatus* (Gaffney, 1979a), but suggest that the triturating surfaces of *Pleurosternon bullockii* have similar proportions to those of UCM 53971 (Evers et al., 2020). The triturating surfaces of UCM 53971 otherwise are narrower than those of *Compsemys victa* (Lyson & Joyce, 2011) and have similar proportions to those of *Dorsetochelys typocardium* (Evans & Kemp, 1976). The foramen supramaxillare is entirely formed by the maxilla and located within the orbit close to the suture with the jugal. Within the maxilla, the foramen supramaxillare leads into the canalis infraorbitalis, which extends anteriorly. A multitude of canals connects the ventral surface of the maxilla to the canalis infraorbitalis along its path. At the level of the ascending process of the maxilla, the canalis alveolaris superior splits from the canalis infraorbitalis. The canalis alveolaris superior, which contained the superior alveolar artery, then extends dorsomedially and exits the maxilla through the foramen alveolare superius to join the fossa nasalis.

#### Vomer

**Fig. 4:**
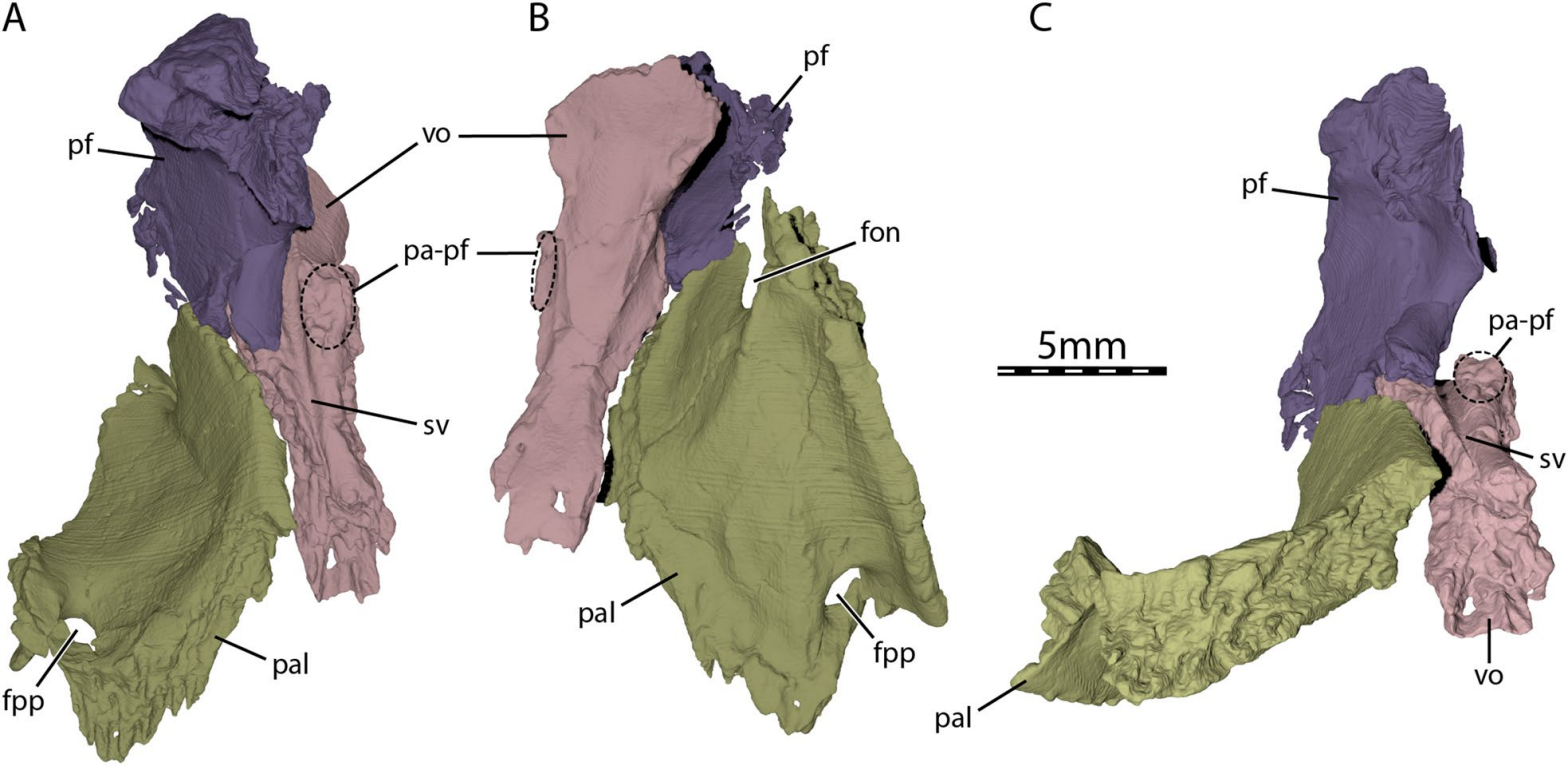
Three-dimensional renderings of the palatal region of UCM 53971. **A** Dorsal view, **B** ventral view, and **C** posterior view. *fon* foramen orbito-nasale, *fpp* foramen palatinum posterius, *pal* palatine, *pa-pf* process for articulation with the prefrontal, *pf* prefrontal, *sv* sulcus vomeri, *vo* vomer

The vomer is a single, narrow bone, which floors the posteromedial part of the fossa nasalis and forms the medial margins of the apertura narium interna (Fig. 2B–C). The vomer is relatively flat, but its anterior part is significantly broadened, similar to the vomer of some pleurodires (Gaffney, 1979b). The vomer contacts the maxilla anterolaterally along a short suture and the palatine posterolaterally along an elongate suture (Figs. 1B and 2A). The vomer reaches the pterygoid posteriorly and prevented the palatine from contacting its counterpart medially (Fig. 1B), as observed in *Compsemys victa* (Lyson & Joyce, 2011) and *Dorsetochelys typocardium* (Evans & Kemp, 1976). At about mid-length, the vomer of UCM 53971 bears extremely low dorsolateral processes for articulation with the prefrontals (Fig. 4). The bar of bone that connects the vomer with the prefrontal is mostly formed by the prefrontal (Fig. 4). This condition is similar to what is observed in testudinoids, but contrasts with trionychids, in which the vomer forms most of this bony connection (Loveridge & Williams, 1957), and pleurodires, in which the vomer–prefrontal contact is absent (Gaffney, 1979b). A similar vomer–prefrontal contact as in UCM 53971 is present in *Compsemys victa* (Lyson & Joyce, 2011), *Glyptops ornatus* (Gaffney, 1979a), and *Pleurosternon bullockii* (Evans & Kemp, 1975), but the extent of this contact is unclear in *Dorsetochelys typocardium* (Evans & Kemp, 1976). The overall shape and contacts of the vomer in UCM 53971 resemble the condition seen in some xinjiangchelyids (see *Annemys levensis*, Rabi et al., 2014) and thalassochelydians (Anquetin & André, 2020; Anquetin et al., 2015, 2017). The sulcus vomeri is a shallow, narrow groove and extends along the posterodorsal part of the vomer and deeply into the interorbital space (Fig. 4A and C), as is the case in a diverse mix of cryptodires (e.g., kinosternoids, testudinoids, *Carettochelys*, some cheloniids; Evers et al., 2019).

#### Palatine

The palatine is a plate-like bone that forms the posterior margin of the apertura narium interna, the posterior margin of the foramen orbito-nasale, and the entire margins of the small foramen palatinum posterius (Figs. 1B and 2A). The portion of the palatine between the foramen orbito-nasale and the foramen palatinum posterius floors the medial aspect of the fossa orbitalis and contacts the maxilla laterally. The palatine contacts the descending process of the prefrontal anterodorsally, the vomer medially, the pterygoid posteromedially and posteriorly, and the jugal posterolaterally lateral to the foramen palatinum posterius (Figs. 1B and 2A). The palatine has minor contributions to the triturating surfaces in the form of a low lingual ridge formed along the contact with the maxilla (Fig. 1B).

#### Pterygoid

**Fig. 5:**
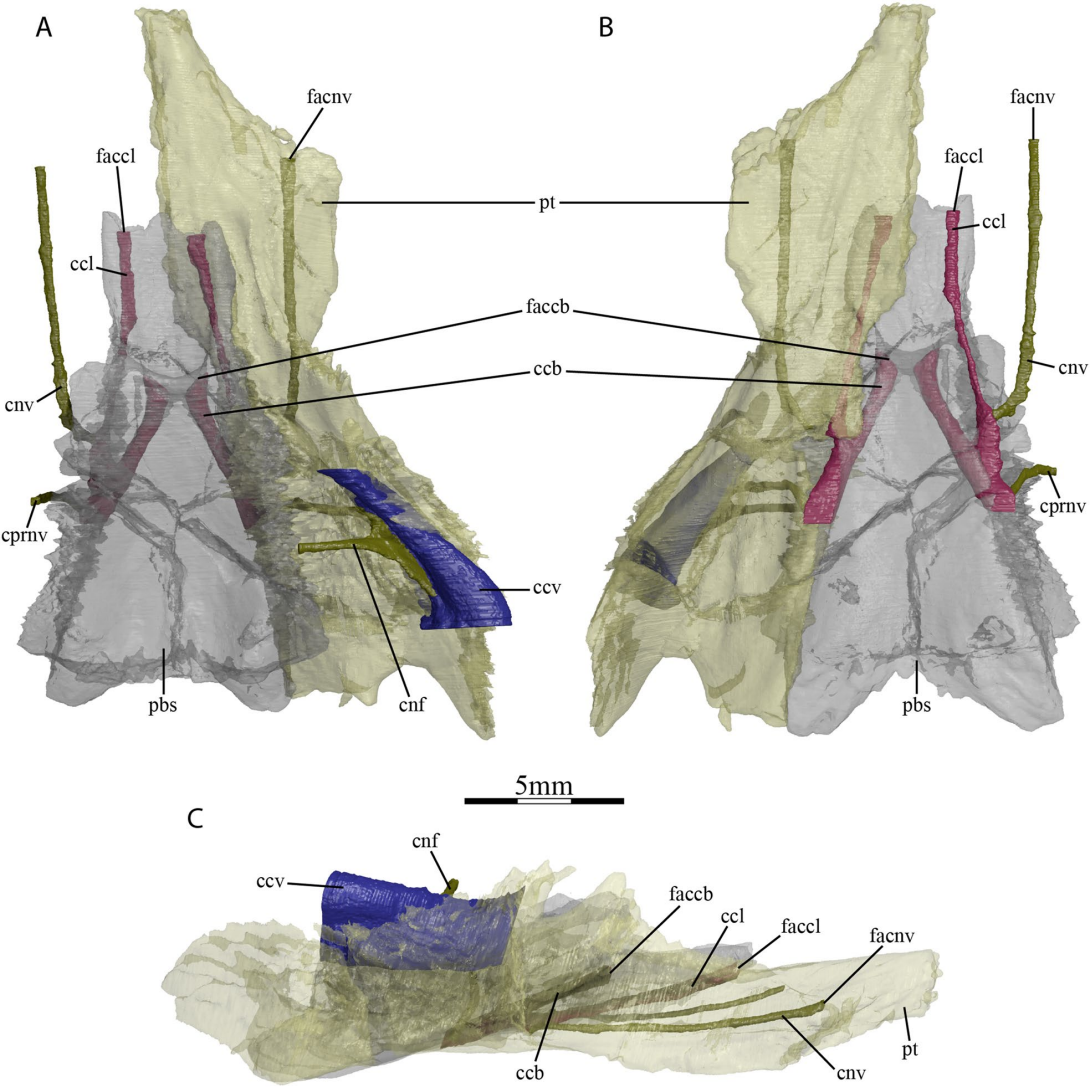
Three-dimensional reconstructions of the parabasisphenoid, right pterygoid, lateral head vein circulation, carotid circulation, and facial nerve system of UCM 53971. **A** Dorsal, **B** ventral, and **C** right lateral view. *ccb* canalis caroticus basisphenoidalis, *ccl* canalis caroticus lateralis, *ccv* canalis cavernosus, *cnf* canalis nervus facialis, *cnv* canalis nervus vidianus, *cprnv* canalis pro ramo nervi vidiani, *faccb* foramen anterius canalis carotici basisphenoidalis, *faccl* foramen anterius canalis carotici lateralis, *facnv* foramen anterius canalis nervi vidiani, *pbs* parabasisphenoid, *pt* pterygoid

**Fig. 6:**
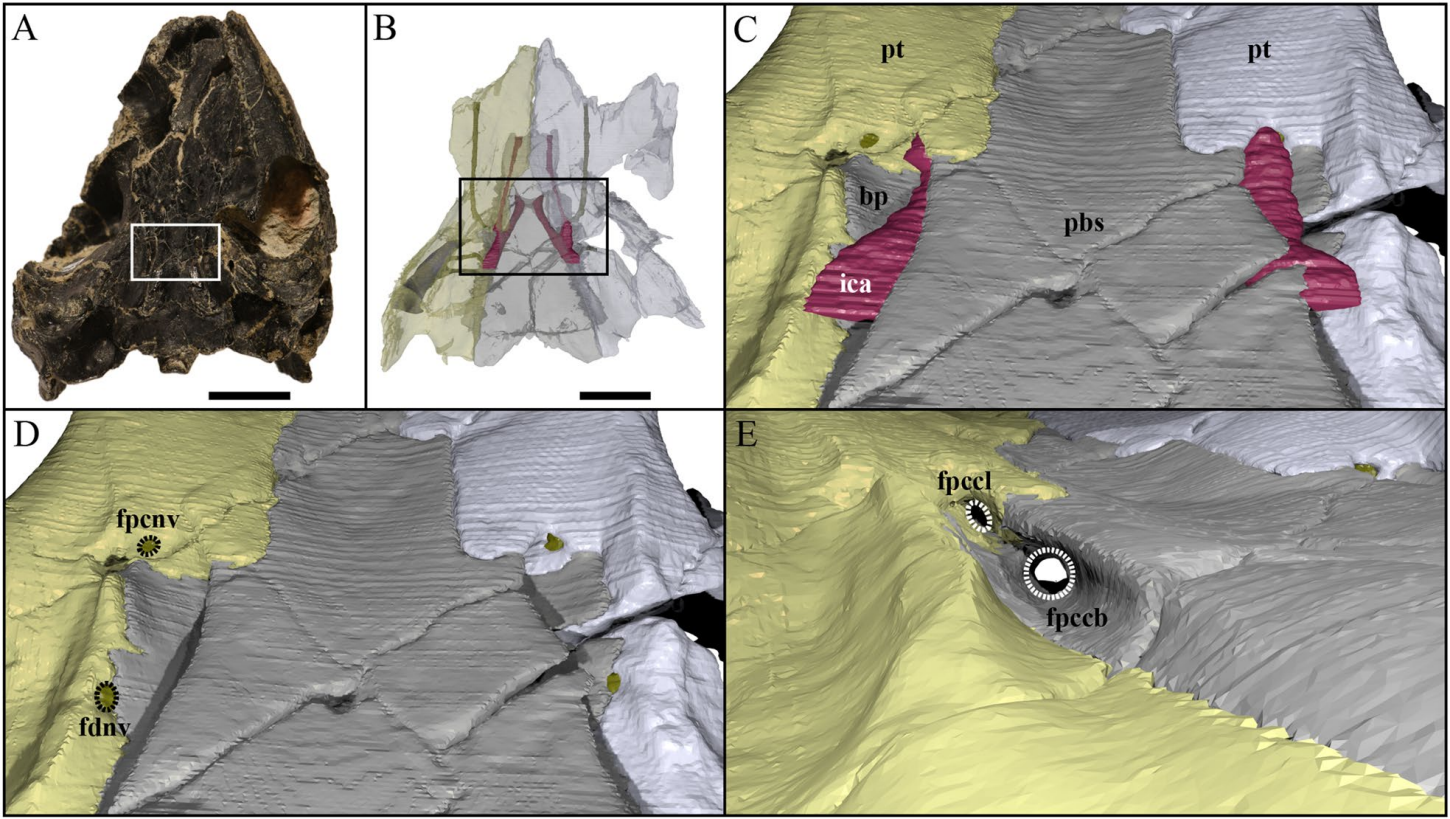
Close-ups on the basicranium and carotid pit of UCM 53971. **A** Indication of the location of the area of interest in the skull, **B** indication of the location of the area of interest relative to Fig. 5, **C** close-up on the basicranium highlighting the path of the internal carotid artery, **D** close-up on the basicranium highlighting foramina for the vidian nerve, and **E** close-up on the right carotid pit highlighting foramina for the carotid circulation system. *bp* basipterygoid process, *fdnv* foramen distalis nervi vidiani, *fpccb* foramen posterius canalis carotici basisphenoidalis, *fpccl* foramen posterius canalis carotici lateralis, *fpcnv* foramen posterius canalis nervi vidiani, *ica* internal carotid artery, *pbs* parabasisphenoid, *pt* pterygoid

The anterior part of the pterygoid contacts its counterpart medially along the midline, the vomer anteromedially, the palatine anteriorly along a V-shaped suture, the maxilla anterolaterally at the posterior end of the triturating surface, and the jugal laterally at the anterior margin of the lower temporal fossa (Fig. 1B). The posterior part of the pterygoid consists of a posterior process that floors the cavum acustico-jugulare and reaches the basioccipital at the same level as the parabasisphenoid (Figs. 1B and 2D). The posterior process of the pterygoid does not extend beyond the posterior limit of the parabasisphenoid (Fig. 1B), as in *Dorsetochelys typocardium* (Evans & Kemp, 1976), *Glyptops ornatus* (Gaffney, 1979a), *Pleurosternon bullockii* (Evans & Kemp, 1975), and *Pleurosternon moncayensis* (Perez-Garcia et al., 2021). The posterior process of the pterygoid contacts the parabasisphenoid posteromedially along a nearly straight suture, the basioccipital posteriorly along a small, concave suture, and the quadrate posterolaterally, but clearly lacks a contact with the exoccipital (Fig. 1B). The pterygoid fossa on the ventral surface of the posterior pterygoid process is moderately deep. The pterygoid possesses a well-developed processus pterygoideus externus that extends posteroventrally into the lower temporal fossa (Fig. 2A). The processus pterygoideus externus of UCM 53971 is dorsoventrally expanded with its lateral surface. The vertical plate of the lateral surface is large, slightly medially recurved along its dorsal margin, and therefore resembles the processus trochlearis pterygoidei of pleurodires, as was recently also observed for *Pleurosternon bullockii* (Evers et al., 2020). A well-developed processus pterygoideus externus is also present in *Glyptops ornatus* (Gaffney, 1979a). The processus pterygoideus externus is anteriorly sutured to the jugal. At mid-length of the suture between the parabasisphenoid and the pterygoid, the pterygoid forms a socket that holds the basipterygoid process of the parabasisphenoid, as in *Glyptops ornatus* (Gaffney, 1979a), *Pleurosternon bullockii* (Evans & Kemp, 1975; Evers et al., 2020), and *Pleurosternon moncayensis* (Perez-Garcia et al., 2021). Jointly with the basipterygoid process of the parabasisphenoid, the pterygoid of UCM 53971 forms a cavity midway along the parabasisphenoid–pterygoid suture, which leads into several foramina dorsally and anteriorly and is posteriorly confluent with a narrow sulcus on the ventral skull surface along the posterior part of the parabasisphenoid–pterygoid suture (Figs. 1B and 5–6). The same morphology has been described for *Pleurosternon bullockii* (Evans & Kemp, 1975; Evers et al., 2020), and grossly similar morphologies seem to be present in *Glyptops ornatus* (Gaffney, 1979a), *Pleurosternon moncayensis* (Perez-Garcia et al., 2021), and *Arundelemys dardeni* (Lipka et al., 2006), but not baenodds (e.g., Gaffney, 1972a; Rollot et al., 2018) or *Compsemys victa* (Lyson & Joyce, 2011). The posterior sulcus present in *Pleurosternon bullockii* and *Uluops uluops* likely held the internal carotid artery, as already proposed by Carpenter and Bakker (1990). The anterior cavity is similar to the fenestra caroticus observed in some turtle groups like xinjiangchelyids, sinemydids, sandownids, or plesiochelyids (Evers & Joyce, 2020; Rabi et al., 2013b; Raselli & Anquetin, 2019), the main difference in *Pleurosternon bullockii* and *Uluops uluops* to the aforementioned turtles being that the posterior course of the internal carotid artery is not encased in bone, but ventrally open. The following foramina can be identified within the anterior region of the cavity of UCM 53971: the foramen posterius canalis nervi vidiani of the facial nerve system (Rollot et al., 2018) and two foramina for the carotid arterial branches, namely the foramen posterius canalis carotici lateralis, and the foramen posterius canalis carotici basisphenoidalis (see Nomenclature above regarding terminology; Fig. 6D–E). Additionally, the foramen distalis nervi vidiani can be identified at the posterior end of the cavity (see below). The foramen posterius canalis nervi vidiani is located on the anterolateral margin of the cavity, anterior to the basipterygoid process (Fig. 6D). It can be unambiguously identified based on the course of the attached canal, the canalis nervus vidianus, which extends anteriorly through the pterygoid and exits the skull through a foramen positioned close to the anterior suture with the palatine, anteroventrolaterally to the epipterygoid (Rollot et al., 2018; Fig. 3). *Uluops uluops* shows unequivocal evidence of osteological correlates for both subordinate branches of the internal carotid artery that are commonly recognized in turtles (Rabi et al., 2013b; Rollot et al., 2021). In addition to the foramen posterius canalis carotici basisphenoidalis, the entrance foramen for the cerebral artery which is located on the medial margin of the cavity within the parabasisphenoid (see Parabasisphenoid) and universally present in turtles, UCM 53971 also shows evidence for the presence of a canal and associated foramina for the palatine artery. The foramen posterius canalis carotici lateralis is located on the anteromedial margin of the cavity and leads to the canalis caroticus lateralis that transmits the palatine branch of the internal carotid artery until its exit foramina within the cavum cranii, the foramen anterius canalis carotici lateralis (Figs. 5 and 6E). This identification is unequivocally supported by the separate coexistence of canals for the vidian nerve and palatine artery (see also Rollot et al., 2021). The pterygoid of UCM 53971 forms the ventral aspects of the foramen posterius canalis carotici lateralis, canalis caroticus lateralis, and foramen anterius canalis carotici lateralis. A summary of apparent variation to the presence of the palatine canal in paracryptodires is included below (see Discussion). A small foramen pierces the pterygoid within the internal carotid groove of UCM 53971, along the parabasisphenoid–pterygoid suture (Fig. 6D). This is identified as the foramen distalis nervi vidiani, which is the ventromedial exit foramen of the posterior portion of the vidian nerve, which is transmitted from the geniculate ganglion to the ventral surface of the skull via the canalis pro ramo nervi vidiani. The foramina for the vidian nerve along the carotid groove and cavity imply that a short section of the vidian nerve was transmitted together with the internal carotid artery in a ventrally exposed position.

**Fig. 7:**
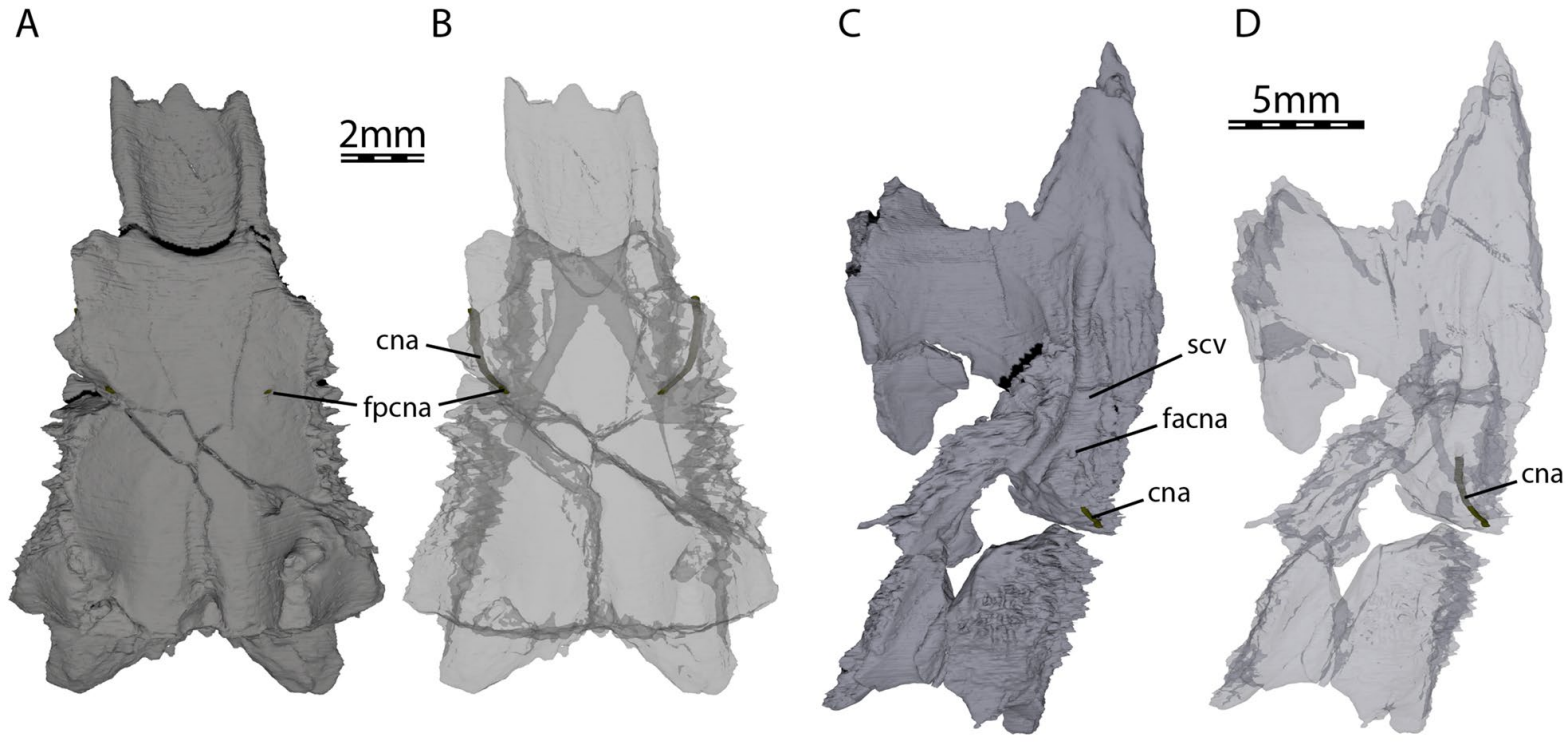
Three-dimensional renderings of the parabasisphenoid, left pterygoid, and canalis nervus abducentis of UCM 53971. **A** Dorsal view of the parabasisphenoid, **B** dorsal view of the transparent parabasisphenoid highlighting the path of the abducens nerve, **C** dorsal view of the left pterygoid, and **D** dorsal view of the transparent left pterygoid highlighting the path of the abducens nerve. *cna* canalis nervus abducentis, *facna* foramen anterius canalis nervi abducentis, *fpcna* foramen posterius canalis nervi abducentis, *scv* sulcus cavernosus

At its posteromedial end, the pterygoid contributes to the formation of anterior tuberculum basioccipitale, which is otherwise mainly formed by the parabasisphenoid (see Parabasisphenoid; Fig. 1B). Within the cavum acustico-jugulare, the pterygoid contacts the prootic anterodorsomedially, the opisthotic dorsomedially, and the quadrate laterally. The pterygoid forms the ventral and anterolateral margins of the canalis cavernosus that extends posteriorly from the foramen cavernosum to the cavum acustico-jugulare, and the ventral and lateral margins of the foramen cavernosum and sulcus cavernosus. The sulcus cavernosus is well defined as a deep groove between the parabasisphenoid medially and the crista pterygoidei of the pterygoid laterally. The crista pterygoidei forms a relatively low crest, which forms a pointed dorsal projection along the anterior margin of the trigeminal foramen, but becomes anteriorly low. In this anterior region of the crista, the pterygoid contacts the epipterygoid anterolaterally along a nearly vertical suture. The pterygoid also frames the ventral and posterior margins of the trigeminal foramen, with a small dorsal process that contacts the parietal in the posterodorsal margin of the trigeminal foramen (Fig. 2B). This parietal–pterygoid contact excludes the prootic from contributing to the trigeminal foramen, as in *Pleurosternon bullockii* (Evers et al., 2020) and *Pleurosternon moncayensis* (Perez-Garcia et al., 2021). The exclusion of the prootic from the trigeminal foramen has also been reported for several baenids (e.g., Brinkman & Nicholls, 1991; Lyson & Joyce, 2009a). However, this is the only contact between the pterygoid and parietal in UCM 53971, as the epipterygoid fully separates the two along the dorsal margin of the crista pterygoidei. Posterior to the trigeminal foramen, the pterygoid contacts the quadrate posteriorly and the prootic posterodorsally (Fig. 2B). The foramina and canalis nervus abducentis have an unusual topological arrangement in UCM 53971. The foramen posterius canalis nervi abducentis is located on the anterodorsal surface of the parabasisphenoid, as in all turtles (Fig. 7A). In all other turtles that we are aware of, the canalis nervus abducentis then traverses the parabasisphenoid in a roughly anterior trajectory, to exit on the anterior surface of the parabasisphenoid, usually ventrally or ventrolaterally to the clinoid processes (e.g., Gaffney, 1979b). In UCM 53971, however, the canalis nervus abducentis extends anterolaterally through the parabasisphenoid, then enters the pterygoid and connects with the posterior portion of the sulcus cavernosus through the foramen anterius canalis nervi abducentis (Fig. 7). Thus, the foramen anterius canalis nervi abducentis is entirely formed by the pterygoid and located in the floor of the sulcus cavernosus posterolateral to the base of the clinoid process of the parabasisphenoid (Fig. 7B–C). The contribution of the pterygoid to the formation of the canalis nervus abducentis and foramen anterius canalis nervi abducentis is highly unusual among turtles as the foramina and canalis nervus abducentis are in most cases entirely formed by the parabasisphenoid. The foramen anterius canalis nervi abducentis is also generally located just lateral to the base of the clinoid process and medial to the sulcus cavernosus (Gaffney, 1979b), although generally close to the pterygoid–parabasisphenoid suture (Anquetin et al., 2015). A few exceptions to this conformation have been highlighted in some taxa. The thalassochelydians *Plesiochelys* and *Portlandemys* have a foramen anterius canalis nervi abducentis in a more posteroventral position relative to the base of the clinoid process than usually observed in turtles (Anquetin et al., 2015; Gaffney, 1976). The condition observed in UCM 53971 is also similar to the baenid *Eubaena cephalica*, which has the lateral margin of the foramen anterius canalis nervi abducentis formed by the pterygoid (Rollot et al., 2018), but differs from *Pleurosternon bullockii*, in which the foramen anterius canalis nervi abducentis is located in the parabasisphenoid within the retractor bulbi pits ventral to the clinoid process (Evers et al., 2020).

#### Epipterygoid

The epipterygoid is a triradiate element that forms the anteroventrolateral wall of the cavum cranii and the anterior margin of the foramen nervi trigemini (Fig. 2B). The epipterygoid contacts the pterygoid ventrally along its entire ventral surface, has a short contact posteriorly with the epipterygoid process of the quadrate, and is tightly sutured with the descending process of the parietal along the dorsal process of the epipterygoid (Fig. 1B). Whereas the epipterygoid is essentially a flat element in *Pleurosternon bullockii* (Evers et al., 2020), the epipterygoid of UCM 53971 has a lateral bulge at its dorsal process, just anterior to the dorsal margin of the trigeminal foramen. The bulge extends as a thick ridge over nearly the entire lateral surface of the epipterygoid, paralleling the anteroventral margin of the obliquely oriented trigeminal foramen. Anterior to the trigeminal foramen, the epipterygoid fully separates the parietal and pterygoid, and is therefore firmly integrated into the secondary lateral braincase wall. In many cryptodires and thalassochelydians (e.g., Evers & Joyce, 2020), the epipterygoid is a more surficial element that only covers the lateral surface of the crista pterygoidei, thereby allowing a broad contact between the crista with the parietal. An ossified epipterygoid is present in *Compsemys victa* (Lyson & Joyce, 2011), *Dorsetochelys typocardium* (Evans & Kemp, 1976), *Glyptops ornatus* (Gaffney, 1979a), *Pleurosternon bullockii* (Evans & Kemp, 1975), and *Pleurosternon moncayensis* (Perez-Garcia et al., 2021), but is absent in baenodds (Gaffney, 1972a).

#### Quadrate

**Fig. 8:**
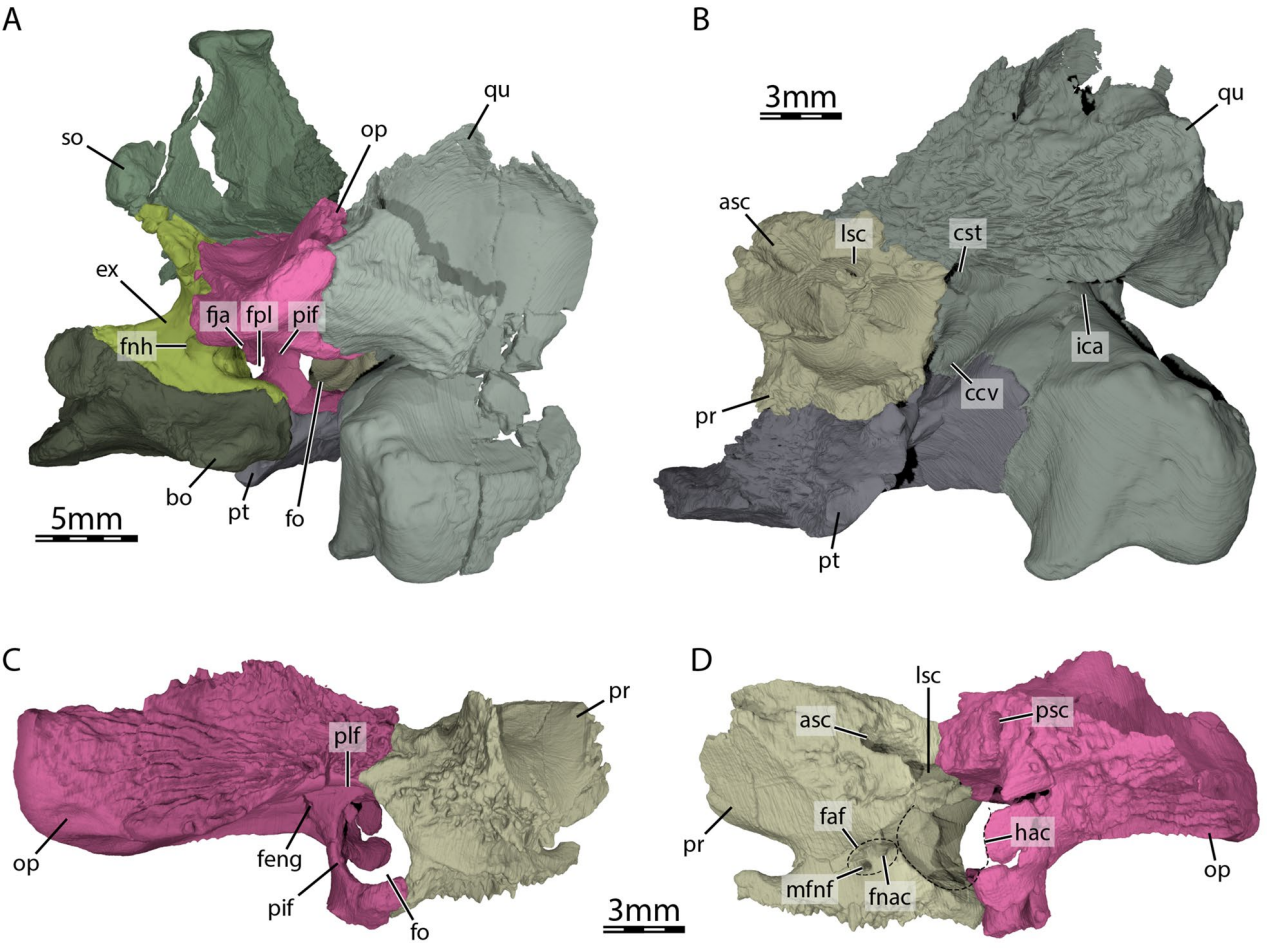
Three-dimensional renderings of the cavum acustico-jugulare and the inner ear capsule of UCM 53971. **A** Posterolateral view of the basicranium and right cavum acustico-jugulare, **B** posterior view of right prootic, pterygoid, and quadrate, **C** lateral view of right prootic and opisthotic, and **D** medial view of right prootic and opisthotic. *asc* anterior semicircular canal, *bo* basioccipital, *ccv* canalis cavernosus, *cst* canalis stapedio-temporalis, *ex* exoccipital, *faf* fossa acustico-facialis, *feng* foramen externum nervi glossopharyngei, *fja* foramen jugulare anterius, *fnac* foramen nervi acustici, *fnh* foramen nervi hypoglossi, *fo* fenestra ovalis, *fpl* fenestra perilymphatica, *hac* hiatus acusticus, *ica* incisura columella auris, *lsc* lateral semicircular canal, *mfnf* medial foramen nervi facialis, *op* opisthotic, *pif* processus interfenestralis of the opisthotic, *plf* perilymphatic fossa, *pr* prootic, *psc* posterior semicircular canal, *pt* pterygoid, *qu* quadrate, *so* supraoccipital

The quadrate forms the condylus mandibularis below the cavum tympani and most of the middle ear, including most of the cavum tympani. In lateral view, the quadrate contacts the quadratojugal anteriorly along a curved, convex suture, and the squamosal posterodorsally (Fig. 2A). The condylus mandibularis does not extend deeply below the cavum tympani, as in all non-baenid paracryptodires. The cavum tympani is a deep cavity that is fully confluent with the antrum postoticum. The quadrate only contributes to its anterodorsal margin. Within the lower temporal fossa, the quadrate forms a short epipterygoid process, which contacts the epipterygoid anteromedially (Fig. 2B). On the dorsal surface of the otic capsule, the quadrate contacts the prootic anteromedially, the supraoccipital medially along a short suture directly posterior to the foramen stapedio-temporale, the opisthotic posteromedially, and the squamosal posterolaterally (Figs. 2B–C and 8A). A short, medial contact with the supraoccipital directly posterior to the foramen stapedio-temporale is preserved on the intact right side of the skull. The quadrate forms the lateral margin of the aditus canalis stapedio-temporalis and canalis stapedio-temporalis, and the posterolateral margin of the canalis cavernosus (Fig. 8B). The foramen stapedio-temporale is exposed on the dorsal surface of the otic capsule (Fig. 2B), and is bordered laterally by the quadrate and medially by the prootic, as in *Pleurosternon bullockii* (Evers et al., 2020) and *Pleurosternon moncayensis* (Perez-Garcia et al., 2021). The foramen stapedio-temporale of *Compsemys victa* is also formed by the supraoccipital in addition to the quadrate and prootic (Lyson & Joyce, 2011). Two facets separated by a shallow sulcus are present on the condylus mandibularis for the articulation with the mandible. The quadrate does not contribute to the foramen nervi trigemini (Fig. 2B). The quadrate contributes to the lateral half of the processus trochlearis oticum and forms the lateral wall of the cavum acustico-jugulare (Fig. 8A–B). The quadrate forms the incisura columella auris (Fig. 8B), which is opened posteroventrally as in *Dorsetochelys typocardium* (Evans & Kemp, 1976), *Glyptops ornatus* (Gaffney, 1979a), *Pleurosternon bullockii* (Evans & Kemp, 1975), and *Pleurosternon moncayensis* (Perez-Garcia et al., 2021).

#### Prootic

The prootic forms the anteromedial portion of the otic capsule. It contacts the parietal anteromedially, the supraoccipital posteromedially, the quadrate laterally, the pterygoid ventrolaterally, the parabasisphenoid ventromedially, and the opisthotic posteriorly (Figs. 2B–C and 8). A small contact between the parietal and pterygoid posterior to the foramen nervi trigemini prevents the prootic from contributing to the margin of the latter (Fig. 2B), as is also the case in *Pleurosternon bullockii* (Evers et al., 2020). The prootic of UCM 53971 is broadly exposed on the dorsal surface of the otic capsule, where it forms the medial half of the foramen stapedio-temporale (Fig. 2B). The medial surface of the canalis stapedio-temporalis is also formed by the prootic, and leads ventrally through the otic capsule into the cavum acustico-jugulare (Fig. 8B). Here, the prootic borders the canalis cavernosus medially, which is the passage between the foramen cavernosum and the cavum acustico-jugulare posteriorly. The foramen cavernosum is also formed laterally by the prootic. The anterior part of the prootic significantly extends anterodorsally beyond the level of the foramen cavernosum, forming a roof-like extension above the trigeminal area including the cavum epiptericum for the trigeminal ganglion. Medially to the cavum epiptericum, the ventral base of the prootic that rests against the parabasisphenoid forms a deep notch for the trigeminal nerve stem (see Evers et al., 2019). Additionally, the prootic forms the anterior parts of the lateral wall of the cavum cranii, and forms the fossa acustico-facialis on its medial margin at the interface with the cavum cranii (Fig. 8D). From this fossa, the canals of the facial nerve (VII) or canalis nervus facialis and for the acoustic nerve (VIII) enter the prootic (Fig. 8D). The canal for the acoustic nerve connects the fossa acustico-facialis to the cavum labyrinthicum and the canalis nervus facialis connects the fossa acustico-facialis to the ventromedial part of the canalis cavernosus (Fig. 3A). The prootic forms the anterior portion of the cavum labyrinthicum, including the anterior halves of the canalis semicircularis anterior and horizontalis (Fig. 8B and D). The foramen aquaducti vestibuli is ossified within the prootic–supraoccipital contact, and anterior to the hiatus acusticus. The prootic and opisthotic form a fully ossified (= surrounded by bone) fenestra ovalis (Figs. 2D and 8C), as also found in *Pleurosternon bullockii* (Evers et al., 2020) and *Pleurosternon moncayensis* (Perez-Garcia et al., 2021). The posterior surface of the prootic just lateral to the fenestra ovalis has a deep perilymphatic fossa (Fig. 8C), as in *Pleurosternon bullockii* (Evers et al., 2020).

#### Opisthotic

The opisthotic is largely exposed in dorsal view through the upper temporal emargination (Fig. 1A). It contacts the supraoccipital anteromedially, the quadrate anterolaterally, the squamosal posterolaterally, the exoccipital posteromedially, the prootic anteriorly, the parabasisphenoid ventromedially, and the pterygoid and basioccipital ventrally along the processus interfenestralis (Figs. 1A, 2D, and 8). The opisthotic does not contribute to the foramen stapedio-temporale and canalis stapedio-temporalis. The posterolaterally and slightly ventrally directed paroccipital process of the opisthotic forms the dorsal margin of the fenestra postotica and roofs the cavum acustico-jugulare (Fig. 2D and Fig. 8A). The dorsal surface of the paroccipital process bears a prominent ridge posteromedially, which bounds an elongated fossa or groove that parallels the posteromedial margin of the process. The cavum acustico-jugulare is separated from the cavum labyrinthicum by the processus interfenestralis (Fig. 8A and C). The processus interfenestralis contacts the basioccipital and pterygoid ventrally along a horizontal suture. The ventral base of the processus interfenestralis is anteriorly expanded to a slight footplate, which also contacts the prootic in the ventral margin of the fenestra ovalis, which is notably large in UCM 53971 (Fig. 8C). Within the cavum labyrinthicum, the opisthotic forms canals for the posterior and lateral semicircular ducts (Fig. 8D). The processus interfenestralis furthermore delimits the recessus scalae tympani anteriorly and forms the lateral and dorsal margins of the fenestra perilymphatica within the recess (Fig. 8A). Also within the recessus scalae tympani, the opisthotic forms the anterior margin of the foramen jugulare anterius. A tiny ventromedial contact with the parabasisphenoid is present along the processus interfenestralis, as in *Pleurosternon bullockii* (Evers et al., 2020) and *Pleurosternon moncayensis* (Perez-Garcia et al., 2021), and an additional contact with this bone is observed posteriorly to the hiatus acusticus. The foramen externum nervi glossopharyngei (IX) and foramen internum nervi glossopharyngei are visible at the dorsal base of the processus interfenestralis (Fig. 8C).

#### Supraoccipital

The supraoccipital is a singular, unpaired element that forms the posterior tip of the skull roof, roofs the cavum cranii posteriorly, and forms the dorsal margin of the foramen magnum (Figs. 1A and 2D). The supraoccipital forms a large, vertical sheet of bone between the skull roof and foramen magnum. The great ventrodorsal depth of the adductor fossa/upper temporal fossa is similar to that seen in early turtles like *Australochelys africanus*, but the supraoccipital is mediolaterally thickened significantly within the fossa in these turtles. Posteriorly, the crista supraoccipitalis of UCM 53971 is short and barely protrudes beyond the foramen magnum (Fig. 1A). The supraoccipital is slightly exposed dorsally in the skull roof, forming a quadrangular surface that contacts the parietals anteriorly (Fig. 1A). A similar exposure is present in *Dorsetochelys typocardium* (Evans & Kemp, 1976), but not in *Pleurosternon bullockii* (Evans & Kemp, 1975; Evers et al., 2020), in which the dorsal exposure of the supraoccipital is much smaller. Within the floor of the upper temporal fossa, the supraoccipital contacts the parietal anteriorly, the prootic anterolaterally, the opisthotic posterolaterally, and the exoccipital posteriorly (Figs. 1A, 2B, and D). Along its most lateral extension, the undamaged right side of the supraoccipital has a short contact with the quadrate just posterior to the foramen stapedio-temporale, to which it does not contribute. The supraoccipital roofs the cavum labyrinthicum, forms the dorsal margin of the hiatus acusticus, the posterior portion of the canalis semicircularis anterior and the anterior portion of the canalis semicircularis posterior, and borders the foramen aquaducti vestibuli dorsally.

#### Basioccipital

The basioccipital of UCM 53971 is a single, unpaired element developed as a roughly rectangular block in the floor of the cavum cranii, as in other non-baenodd paracryptodires (Fig. 1B). The basioccipital forms the ventral margin of the foramen magnum and the complete articular surface of the condylus occipitalis as in *Dorsetochelys typocardium* (pers. comm. by Jérémy Anquetin about the holotype DORCM G.23), *Pleurosternon moncayensis* (Perez-Garcia et al., 2021), and very likely *Glyptops ornatus* (Gaffney, 1979a). The basioccipital contacts the exoccipitals dorsally along a horizontal suture (Fig. 2D). A low crista dorsalis basioccipitalis is present on the anterior portion of the dorsal basioccipital surface. Posterolaterally, the basioccipital bears a short, but thick tuberculum basioccipitale, which is formed without contributions of any other bones (Fig. 1B). The basioccipital is significantly broader at the level of the tubercula basioccipitale than the parabasisphenoid. Within the cavum acustico-jugulare, the basioccipital contacts the processus interfenestralis of the opisthotic anterodorsally along a horizontal suture. The basioccipital contacts the parabasisphenoid anteriorly along a convex suture, which is laterally framed by the anterior tubercula basioccipitale that are jointly formed by the parabasisphenoid and pterygoids, and overlap parts of the ventral basioccipital surface (Fig. 1B). The basioccipital forms a slight depression posterior to its contact with the parabasisphenoid and two foramina basioccipitale are located on the ventral surface of the basioccipital within this depression.

#### Exoccipital

The exoccipital contacts the supraoccipital dorsomedially, the opisthotic dorsolaterally, and the basioccipital ventrally (Figs. 1A and 2D). A contact with the pterygoid is clearly absent. The exoccipital forms the posterolateral wall of the cavum cranii, the lateral margin of the foramen magnum, the medial margin of the fenestra postotica, the posterior margin of the foramen jugulare anterius, and roofs the cavum acustico-jugulare posteromedially. Within the cavum acustico-jugulare, the exoccipital contacts the opisthotic anteriorly and forms the medial and ventral margins of the fenestra perilymphatica and the posterior margin of the foramen jugulare anterius (Fig. 8A). The exoccipital bears three foramina nervi hypoglossi (XII) that gradually increase in size towards the posterior (Figs. 2D and 8A). Only two foramina nervi hypoglossi are present in *Dorsetochelys typocardium* (Evans & Kemp, 1976), *Glyptops ornatus* (Gaffney, 1979a), and *Pleurosternon moncayensis* (Perez-Garcia et al., 2021). The anteriormost two foramina nervi hypoglossi already lie within the cavum acustico-jugulare, a condition observed for several relatively basal turtles, including meiolaniforms, *Eileanchelys waldmani*, or *Kallokibotion bajazidi* (Evers & Benson, 2019). The exoccipital reaches onto the dorsolateral part of the condylus occipitalis, but does not contribute to the condylar articular facet for the atlas (Figs. 2D and 8A).

#### Parabasisphenoid

The parabasisphenoid is a single, unpaired element exposed on the ventral surface of the skull that forms most of the ventral margin of the cavum cranii. The parabasisphenoid contacts the pterygoid laterally along its entire length, the prootic dorsolaterally, and the basioccipital posteriorly along a straight vertical suture (Fig. 1B). An additional, small contact with the opisthotic is present at the posterolateral end of the parabasisphenoid. The parabasisphenoid forms the rostrum basisphenoidale, the sella turcica, the dorsum sellae, and the processus clinoideus. The parabasisphenoid is dorsally concave from the dorsum sellae to its posterior end. This concavity is interrupted along the midline by a high crista basis tuberculi basalis. The rostrum basisphenoidale is a narrow sheet of bone anterior to the dorsum sellae that covers the anterior portion of the canalis caroticus lateralis and forms the dorsal margin of the foramina anterius canalis carotici lateralis (Fig. 5A–B). Thus, the position of these foramina is somewhat unusual, in that they are positioned more medially than in most turtles, where the anterior exiting foramina are situated within the pterygoid–parabasisphenoid suture along the floor of the sulcus cavernosus, and thus lateral, rather than ventral to the rostrum basisphenoidale. The sella turcica is a moderately deep depression that contains the foramina anterius canalis carotici basisphenoidalis, which are relatively widely spaced (Fig. 5A–B). The dorsum sellae overhangs the sella turcica anteriorly, but there is no vertical ridge on the anterior surface below the dorsum sellae. The processus clinoideus are partially damaged, but the preserved parts indicate that they were likely relatively short and had broad bases. Posteriorly, the bases of the clinoid processes form together with the prootic a relatively deep notch that would have allowed the trigeminal nerve stem to pass from the cavum cranii into the cavum epiptericum (Evers et al., 2019). The broad base of the clinoid processes also overhangs the sulcus cavernosus anterolaterally, almost contacting the secondary lateral braincase wall that is formed by the epipterygoid at this level. This morphology hypertrophies the depth of the retractor bulbi pits ventrally to the clinoid processes, on the surface that rises vertically medial to the sulcus cavernosus. Usually, the anterior abducentis nerve foramina are positioned in this surface. In UCM 53971, these foramina are unusual in that they are more laterally placed than seen in other turtles within the floor of the sulcus cavernosus (Fig. 7C). UCM 53971 is furthermore the only turtle of which we are aware that has these abducentis foramina positioned within the pterygoid, rather than the parabasisphenoid (Fig. 7C–D). The abducens canal can be traced posteriorly from these foramina through the pterygoid and then the parabasisphenoid, where the posterior foramina are located in their ‘regular’ positions within the cup-shaped, dorsal parabasisphenoid surface. The parabasisphenoid forms most of the medial margin of the sulcus cavernosus and the ventral margin of the hiatus acusticus and has a slight posterolateral contact with the processus interfenestralis of the opisthotic. At about midpoint along the parabasisphenoid–pterygoid suture on the ventral skull surface, the parabasisphenoid has a laterally projecting basipterygoid process on each side, which extends laterally and inserts into a respective socket formed by the pterygoid (Fig. 6C–D). The overall morphology of this area is very similar to that of *Pleurosternon bullockii* (Evers et al., 2020) and *Pleurosternon moncayensis* (Perez-Garcia et al., 2021), with differences mainly in the presence vs. absence of a palatine artery (see Pterygoid above). At the level of the basipterygoid process, the parabasisphenoid and pterygoid form a cavity that is mainly bordered by the basipterygoid process of the parabasisphenoid (Figs. 1B and 6C–D), which is also present in *Glyptops ornatus* (Gaffney, 1979a) and *Pleurosternon bullockii* (Evans & Kemp, 1975; Evers et al., 2020). The vidian nerve as well as the palatine and cerebral arteries enter the skull through this cavity, but only the foramen posterius canalis carotici basisphenoidalis is formed by the parabasisphenoid (Fig. 6D–E). Posterior to the cavity located along the parabasisphenoid–pterygoid suture is a groove that extends posteriorly and is mainly formed by the parabasisphenoid (Fig. 6C–E). This groove housed the internal carotid artery. The parabasisphenoid, along with minor lateral contributions of the pterygoid, forms posterior processes on each side that overlap the basioccipital posteroventrally (Fig. 1B). These have been called anterior tubercula basioccipitale (Evers et al., 2020), as they are likely homologous with such structures of helochelydrids (Joyce et al., 2014). The anterior tubercula basioccipitale are slightly raised posteriorly in UCM 53971, forming a shallow fossa between them. In addition to helochelydrids, the anterior tubercula basioccipitale are also present in *Glyptops ornatus* (Gaffney, 1979a), *Pleurosternon bullockii* (Evans & Kemp, 1975; Evers et al., 2020), *Pleurosternon moncayensis* (Perez-Garcia et al., 2021), and *Dorsetochelys typocardium* (DORCM G.00023).

#### Labyrinth morphology

**Fig. 9:**
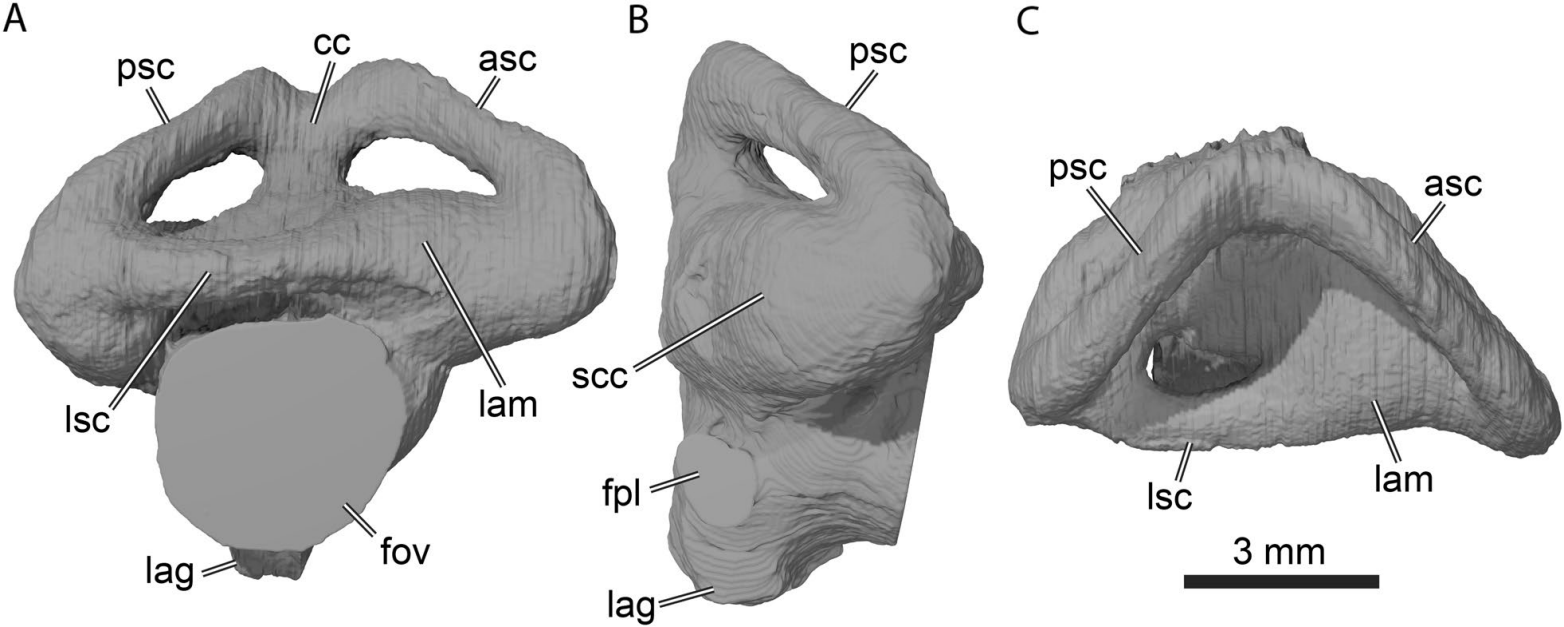
3D rendering of the right endosseous labyrinth of UCM 53971. **A** Lateral view, **B** posterior view, **C** dorsal view. *asc* anterior semicircular canal, *cc* common crus, *fov* fenestra ovalis, *fpl* fenestra perilymphatica, *lag* lagena, *lam* lateral ampulla, *lsc* lateral semicircular canal, *psc* posterior semicircular canal, *scc* secondary common crus

The labyrinth of UCM 53971 (Fig. 9) conforms to the general morphology of turtle labyrinths (Evers et al., 2019; Lautenschlager et al., 2018), but notably is morphologically distinct from other paracryptodire labyrinths that have been described (Pérez-García et al. 2021; Evers et al., 2021). The endosseous labyrinth of *Uluops uluops* is formed by the prootic, opisthotic, and supraoccipital. The anterior semicircular canal is slightly longer than the posterior semicircular canal (Fig. 9A and C). Both vertical semicircular canals are straight along their central sections, and most of the curvature is achieved near the ampullae and the common crus, which is dorsally embayed between the tallest section of the anterior and posterior semicircular canals (Fig. 9A). This dorsal embayment contrasts with the morphology of the labyrinth of *Arundelemys dardeni*, which lacks a clear embayment (Evers et al., 2021). The strongest difference between the labyrinth of *U. uluops* with other paracryptodires, notably the pleurosternid *Pleurosternon moncayensis* (Pérez-García et al. 2021), is the relative thickness of the semicircular canals, which is high in *U. uluops*, whereas other paracryptodires have slender canals. Pérez-García et al. (2021) interpret the slender semicircular canals of *P. moncayensis* as indicative of freshwater habits of pleurosternids, but the thick canals in *U. uluops* and its ecological interpretation as a freshwater turtle show that labyrinth morphology may be a poor indicator of ecology, especially when interpreted without a comprehensive comparative sample (e.g., Bronzati et al., 2021).

The angle between the vertical semicircular canals is roughly 90° in *Uluops uluops* (Fig. 9C), which is similar to the angle reported for *Pleurosternon moncayensis* (Pérez-García et al. 2021). The lateral and anterior ampullae of *U. uluops* are not well delimited from one another, and the ventral section of the posterior semicircular canal and the posterior section of the lateral semicircular canal are confined to a single osseous cavity, the secondary common crus (Fig. 9B). The fenestra ovalis is a large opening (Fig. 9A) that is fully closed by the prootic and opisthotic. The fenestra perilymphatica is also well defined and completely closed (Fig. 9B), therefore not leaving a hiatus postlagenum. Unlike in most turtles including other paracryptodires (Pérez-García et al. 2021; Evers et al., 2021), the ventral section of the endosseous labyrinth is well defined by bone, so that the lagena part of the labyrinth housing the cochlea is formed as a small ventral protrusion between the opisthotic, basioccipital and parabasisphenoid (Fig. 9A–B).

#### Stapes

The stapes is not preserved in UCM 53971.

#### Internal carotid artery circulation pattern

The internal carotid artery is ventrally exposed in *Uluops uluops*, albeit funneled along a ventrally open groove that extends nearly antero-posteriorly along parts of the parabasisphenoid–pterygoid suture, as observed by Carpenter and Bakker (1990). As the internal carotid artery is never fully enclosed by bone, a canalis caroticus internus and foramen posterius canalis carotici interni are absent. The internal carotid groove ends anteriorly in a pit that resembles the fenestra caroticus of xinjiangchelyids in its topological position and by housing the splitting point of the internal carotid artery (Rabi et al., 2013b), but differs by being posteriorly open and connected to the carotid groove (Fig. 6C–E). The carotid pit of *Uluops uluops* is mainly formed by the basipterygoid process of the parabasisphenoid and located midway along the parabasisphenoid–pterygoid suture (Figs. 1B and 6C–E). Although paracryptodires were previously believed to universally possess a palatine artery (e.g., Brinkman & Nicholls, 1993; Gaffney, 1982), newer studies based on µCT data suggest that the artery is generally absent (Lipka et al., 2006; Rollot et al., 2018; Evers et al., 2020, 2021; Pérez-Garcia et al., 2021). *Uluops uluops* is the only paracryptodire with µCT data available that shows clear evidence that its internal carotid artery splits into both a medially branching cerebral and anterolaterally branching palatine artery. Reports that the canal is also present in *Dorsetochelys typocardium* (Anquetin & André, 2020) require verification with µCT data. The foramina posterius canalis carotici lateralis are located within the carotid pit and can be traced forward through their canals and anterior foramina (Fig. 6E). The canalis caroticus lateralis is long, has a small diameter, and extends anteriorly along the parabasisphenoid–pterygoid suture (Fig. 5). The anterior portion of the canalis caroticus lateralis is covered by the rostrum basisphenoidale. The foramen anterius canalis carotici lateralis is located at the anterior end of the rostrum basisphenoidale and formed by the pterygoid ventrally and the parabasisphenoid dorsally (Fig. 5A–B). The canalis caroticus basisphenoidalis extends anteromedially within the parabasisphenoid and enters the sella turcica by way of the relatively widely spaced foramina anterius canalis carotici basisphenoidalis (Fig. 5A–B).

The palatine artery appears to be greatly reduced compared to the cerebral and stapedial arteries. We measured the cross-sectional areas of the canalis caroticus basisphenoidalis, canalis caroticus lateralis, and canalis stapedio-temporalis close to their exit foramina of the skull, namely the foramen anterius canalis carotici basisphenoidalis, foramen anterius canalis carotici lateralis, and foramen stapedio-temporale, respectively. The surface of the cross section of the canalis caroticus lateralis is 0.090 mm^2^ on the left and 0.096 mm^2^ on the right. The surface of the cross section of the canalis caroticus basisphenoidalis is 0.258 mm^2^ on the left and 0.251 mm^2^ on the right. The surface of the canalis stapedio-temporalis was only measured with confidence on the right side, as the left otic capsule is damaged and some parts of the bones forming the margin of the left canalis stapedio-temporalis are missing. The surface value on the right side is 1.076 mm^2^. These results highlight that the stapedial artery is the largest and that the palatine artery is the smallest among the three canals measured. The palatine artery is less than half the size of the cerebral artery and only one tenth of the size of the stapedial artery, and may have been insignificant for blood supply, therefore potentially explaining its possibly repeated loss in paracryptodires (see Discussion).

#### Canalis cavernosus

The canalis cavernosus is an antero-posteriorly directed canal that contains the lateral head vein and connects the cavum cranii to the cavum acustico-jugulare (Fig. 5). The anterior portion of the canalis cavernosus is formed by the prootic dorsally and dorsomedially and by the pterygoid laterally and ventrally. The posterior portion of the canalis cavernosus is formed by the prootic medially and dorsally, by the pterygoid ventrally, and by the quadrate laterally. The canalis cavernosus can be subdivided into two sections: a ventral one and a dorsal one, separated by a constriction located on the medial side of the canalis cavernosus. The constriction is particularly strong at the level of contact between the canalis cavernosus and canalis nervus facialis, and has been suggested to be an osteological correlate for the separation between the lateral head vein and the mandibular artery (Rollot et al., 2021). In UCM 53971, the lateral head vein is housed in the ventral part of the canalis cavernosus and the mandibular artery in the dorsal part. The canalis cavernosus contacts the canalis nervus facialis along its ventral portion (Fig. 5A).

#### Facial nerve system

The facial nerve extends laterally from the fossa acustico-facialis to the canalis cavernosus through a long and small canalis nervus facialis, which is located in the prootic (Fig. 5A and C). From within the canalis cavernosus, the facial nerve divides into two branches at the geniculate ganglion: the hyomandibular and vidian nerves. A sulcus for the hyomandibular nerve is present and extends posteriorly to the cavum acustico-jugulare within the ventromedial margin of the canalis cavernosus, formed by the prootic and pterygoid (Fig. 5A). The vidian nerve enters the canalis pro ramo nervi vidiani slightly anterior to the contact between the canalis nervus facialis and canalis cavernosus (Fig. 5A–B). The canal is long, and extends ventromedially through the pterygoid, where it exits via the foramen distalis nervi vidiani and into the carotid groove that houses the internal carotid artery (Fig. 6D). The vidian nerve is inferred to first follow the path of the internal carotid artery into the carotid pit, and then the path of the palatine artery for a short distance within this cavity. The palatine artery and vidian nerve courses become separate in the anterior margin of the carotid pit, where they enter separate canals via distinct foramina (Fig. 5B and Fig. 6C–E). The canalis nervus vidianus for the vidian nerve starts lateral to the palatine artery canal, extends anteriorly through the pterygoid, and exits the skull by way of the foramen anterius canalis nervi vidiani, which is located on the dorsal surface of the pterygoid, anteroventrolaterally to the anterior end of the epipterygoid (Fig. 5). A nearly identical facial, hyomandibular, and vidian nerves pattern is present in *Pleurosternon moncayensis* (Perez-Garcia et al., 2021) and *Pleurosternon bullockii* (Evers et al., 2020), the main difference with UCM 53971 being that the vidian nerve does not follow the path of the palatine artery in *Pleurosternon bullockii* as this branch is absent.

#### Canalis basioccipitalis

The canalis basioccipitalis are paired canals that emerge from the ventral surface of the basioccipital by means of the small foramen basioccipitale. Owing to their very small size, the canalis basioccipitalis can only be followed for a very short distance within the basioccipital. The path of the canalis basioccipitalis is unclear but they might merge within the basioccipital.

## Discussion

### Basicranium and circulation system

The clade *Paracryptodira* was originally defined by Gaffney (1975) based on the location of the foramen posterius canalis carotici interni, the posterior entrance of the internal carotid artery into the skull, midway along the parabasisphenoid–pterygoid suture. This feature, which has since universally been confirmed to be present in paracryptodires (Archibald & Hutchison, 1979; Brinkman, 2003; Brinkman & Nicholls, 1991, 1993; Evans & Kemp, 1975, 1976; Gaffney, 1982; Lively, 2015; Lyson & Joyce, 2009a, 2009b, 2010, 2011; Lyson et al., 2016), contrasts with that of pleurodires, where this foramen is exposed on the ventral surface of the skull and formed by a combination of the parabasisphenoid, prootic, and quadrate and with that of crown cryptodires, where the foramen is located in the pterygoid (Gaffney, 1975). Evers et al. (2020) more recently suggested the existence of two morphotypes among paracryptodires. In the first morphotype, a true, anteriorly positioned foramen posterius canalis carotici interni is present, as in *Compsemys victa* and *Eubaena cephalica*, and in the second morphotype, a depression is present that is formed by the basipterygoid process of the parabasisphenoid, as in *Pleurosternon bullockii* and *Uluops uluops*. In either case, these structures serve as the entry point of the internal carotid artery into the skull, which has then been thought to split into its two subordinate branches, the cerebral and palatine arteries (Gaffney, 1972b, 1975, 1979b, 1982). The canalis caroticus lateralis was first identified in paracryptodires by Gaffney (1972a, 1975) who noted its presence in *Glyptops ornatus* and baenids, and subsequent studies confirmed this observation for baenids (Brinkman, 2003; Brinkman & Nicholls, 1991, 1993; Gaffney, 1982) and *Pleurosternon bullockii* (Sterli et al., 2010). The canalis caroticus lateralis was therefore thought to be universally present in paracryptodires and generally identified along with the foramina anterius canalis carotici lateralis in members of that clade. Over the last twenty years, however, several studies revealed through the use of µCT scans the absence of the canalis caroticus lateralis in a subset of paracryptodires, in particular *Arundelemys dardeni* (Evers et al., 2021; Lipka et al., 2006), *Eubaena cephalica* (Rollot et al., 2018), *Pleurosternon bullockii* (Evers et al., 2020), and *Pleurosternon moncayensis* (Pérez-García et al. 2021). This conclusion is particularly relevant for *P. bullockii* and *E. cephalica*, where the canal initially identified as the canalis caroticus lateralis (Gaffney, 1982; Sterli et al., 2010) was revealed to be the canalis nervus vidianus (Evers et al., 2020; Rollot et al., 2018). We therefore here take the opportunity to review the literature in search of similar examples.

Among basal paracryptodires, Gaffney (1979a) suggested the presence of a canal for the palatine artery in *Glyptops ornatus* but was not able to find a foramen anterius canalis carotici lateralis, while Anquetin and André (2020) identified a foramen posterius canalis carotici lateralis in *Dorsetochelys typocardium*, that is located anterolateral to the foramen posterius canalis carotici basisphenoidalis and mainly formed by the pterygoid. As the foramen posterius canalis nervi vidiani of *Uluops uluops* is located just lateral to the foramen posterius canalis carotici lateralis (see Fig. 6) and a correct identification of these structures was only made possible by the use of µCT scans, we cannot exclude the possibility that the canals and foramina identified in *Dorsetochelys typocardium* and *Glyptops ornatus* actually contained the vidian nerve rather than the palatine artery, although the absence of a foramen anterius canalis carotici lateralis in *Glyptops ornatus* and the placement of the purported foramen posterius canalis carotici lateralis within the pterygoid of *Dorsetochelys typocardium* is suggestive for the presence of a vidian canal alone. The identifications of a palatine artery canal in these taxa should be verified with µCT scans and cross-examination of the facial nerve pattern. Within baenids, a canal and foramina for the palatine artery were identified in *Boremys pulchra* (Brinkman & Nicholls, 1991), *Neurankylus eximius* (Brinkman & Nicholls, 1993), and *Plesiobaena antiqua* (Brinkman, 2003). In *Boremys pulchra*, the canalis pro ramo nervi vidiani joins the canalis caroticus internus just anterior to the foramen posterius canalis carotici interni, and the entry foramen for the palatine artery into the canalis caroticus lateralis is located on the lateral surface of the canalis caroticus internus just anterior to this (Brinkman & Nicholls, 1991). This description resembles very much the circulation system of *Eubaena cephalica*, in which the canalis pro ramo nervi vidiani joins the canalis caroticus internus just anterior to the foramen posterius canalis carotici interni. In *Eubaena cephalica*, just anterior to this junction, the vidian nerve exits the canalis caroticus internus along its lateral margin and enters the canalis nervus vidianus (Rollot et al., 2018). It therefore seems plausible that the foramen identified in *Boremys pulchra* just anterior to the contact between the canalis pro ramo nervi vidiani and the canalis caroticus internus actually corresponds to the passage of the vidian nerve from the internal carotid artery canal into the canalis nervus vidianus. In *Neurankylus eximius*, the canalis caroticus internus contacts the canalis pro ramo nervi vidiani just anterior to the foramen posterius canalis carotici interni, and a small foramen is visible just anterior to this (Brinkman & Nicholls, 1993). This small foramen leads to a canal that was identified as the canalis caroticus lateralis. The branching point of this canal, just anterior to the contact between the canalis caroticus internus and canalis pro ramo nervi vidiani, once again, is reminiscent of the condition observed in *Boremys pulchra* and *Eubaena cephalica* for the path of the vidian nerve. This canal is also described and illustrated as fully crossing the pterygoid and not extending along the parabasisphenoid–pterygoid suture, thus being in a more lateral position than where a canalis caroticus lateralis is expected to be, and more likely corresponding to the usual position of a canalis nervus vidianus as seen in *Eubaena cephalica* (Rollot et al., 2018) and described for extant turtles (Rollot et al., 2021). We therefore suggest that the canal identified as the canalis caroticus lateralis by Brinkman and Nicholls (1993) corresponds to the canalis nervus vidianus instead, and postulate the absence of the palatine artery and canalis caroticus lateralis for *Neurankylus eximius*. In *Plesiobaena antiqua*, the canalis caroticus internus contacts the canalis pro ramo nervi vidiani just anterior to the foramen posterius canalis carotici interni, and a canal identified as the canalis caroticus lateralis branches off the canalis caroticus internus anterior to this to extend anteriorly through the pterygoid (Brinkman, 2003). Although no illustration allows to confirm these statements, the description of the circulation system of *Plesiobaena antiqua* is very similar to that of *Neurankylus eximius* and *Eubaena cephalica*. For the same reasons as mentioned above for the circulation system of *Neurankylus eximius*, we therefore consider it more plausible that the canal identified as the canalis caroticus lateralis by Brinkman (2003) actually corresponds to the canalis nervus vidianus and that the canalis caroticus lateralis is absent. The use of µCT scans will be needed to confirm the aforementioned statements and describe in detail the circulation systems of these taxa. However, the ubiquitous absence of a definitive lateral canal in any known baenid, suggests that it is universally absent in the group.

In helochelydrids (retrieved as *Pleurosternidae* in our analysis; see Phylogenetic relationships), the circulation system remains unknown for *Helochelydra nopcsai* and *Aragochersis lignitesta*, but was decribed for *Naomichelys speciosa* (Paulina-Carabajal et al., 2019). Paulina-Carabajal et al. (2019) provided reconstructions of portions of the internal carotid artery and facial nerve systems in the specimen FMNH PR273 but the incomplete nature of its basicranium as well as the likeliness that the circulation pattern of *Naomichelys speciosa* greatly differs from that of other known turtles make any identification and interpretation of the internal carotid artery pattern difficult. Paulina-Carabajal et al. (2019) identified a foramen posterius canalis carotici interni and several carotid branches, but we are not able to confirm the identity of these branches and canals in the same set of scans of that specimen (personal observations, YR and SWE, 2021). Paulina-Carabajal et al. (2019) additionally provided reconstructions of cranial nerves including the facial nerve and its associated branches. We herein confirm the path of the facial nerve in FMNH PR273 and the presence of the canalis pro ramo nervi vidiani in the pterygoid (their branch number 4; see Figs. 2A1, 2A3–2A4, and 3 in Paulina-Carabajal et al., 2019) that transmits the vidian nerve from the canalis cavernosus to the ventral surface of the pterygoid. The foramen distalis nervi vidiani is thus ventrally exposed as suggested by Joyce et al. (2014). As the understanding of the circulation system in helochelydrids remains partially unknown, the study of the cranial internal anatomy of *Helochelydra nopcsai*, for which a well-preserved skull is known (see Joyce et al., 2011), should provide new insights into the internal carotid and facial nerve patterns of this clade.

**Fig. 10:**
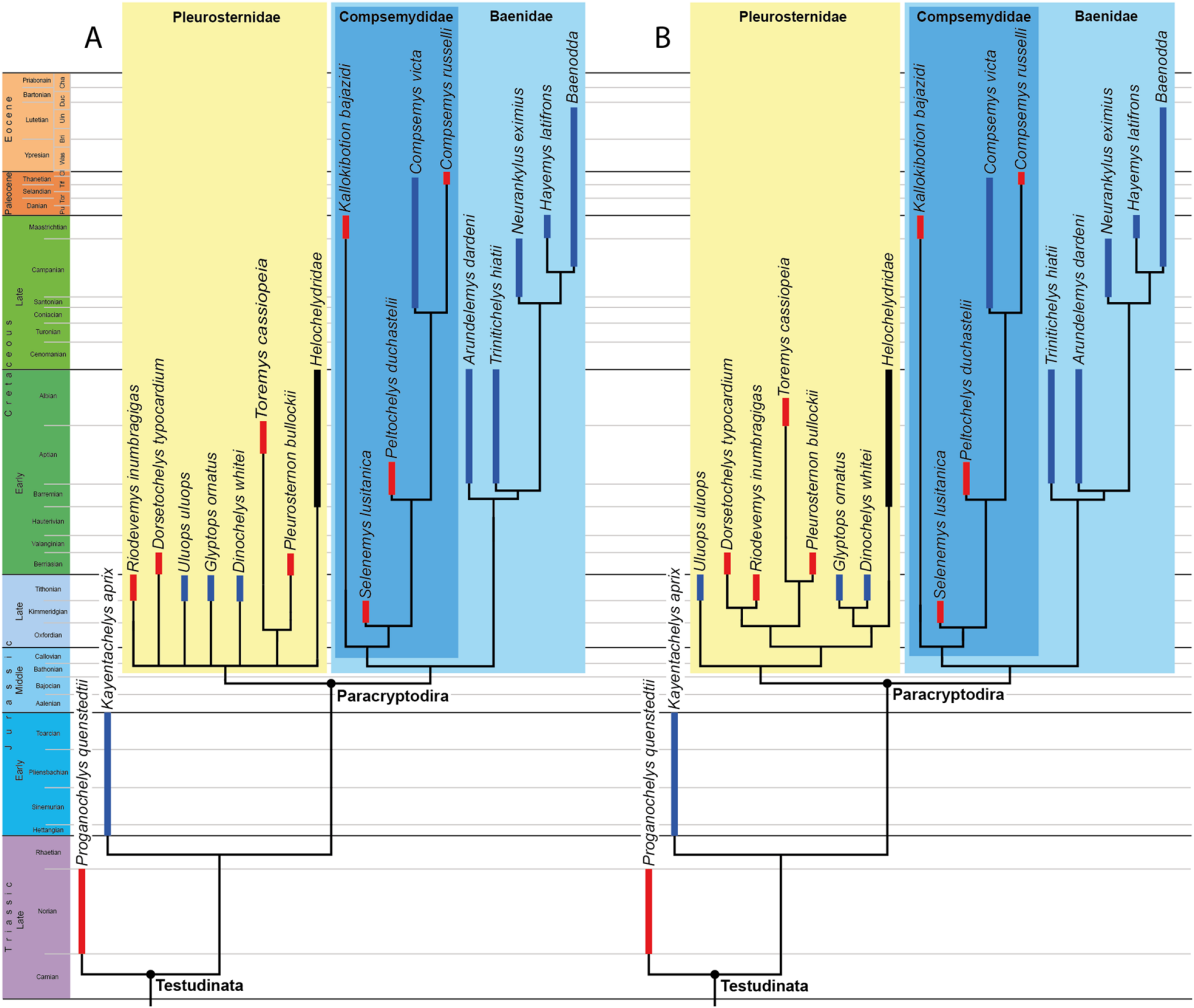
Time-calibrated phylogenetic hypotheses resulting from the expanded analyses of Joyce and Rollot (2020). **A** Strict consensus tree obtained from the analysis under equal weighting without the rogue taxa *Scabremys ornata*, *Cedrobaena putorius*, and *Peckemys brinkman*; **B** single most parsimonious tree obtained from the analysis with implied weighting and a K value of 6 with a best score of 19.55433. The range of North American taxa is highlighted in blue, the range of European taxa in red, and the range of clades that have both a European and North American distribution in black

In conclusion, while the palatine artery and canalis caroticus lateralis were initially inferred to be universally present among paracryptodires, it now appears that the canalis caroticus lateralis can only be identified with confidence in *Kallokibotion bajazidi* (Gaffney & Meylan, 1992; Martín-Jiménez et al., 2021) and *Uluops uluops* (this study). In *Kallokibotion bajazidi*, the internal carotid artery extends extracranially, its split into the cerebral and palatine arteries occurs extracranially, and separate canalis caroticus basisphenoidalis and canalis caroticus lateralis are present (Gaffney & Meylan, 1992; Martín-Jimenez et al. 2021). In *Uluops uluops*, we identified separate canals for the cerebral and palatine arteries, and our interpretations are sound because all expected facial nerve correlates have been identified alongside the canalis caroticus basisphenoidalis and canalis caroticus lateralis. The palatine artery and canalis caroticus lateralis are thereby only present with confidence in the most basal pleurosternid and the most basal compsemydid taxa (see Fig. 10), which likely suggests at least two independent losses of the palatine artery within paracryptodires, even though the placement of *Kallokibotion bajazidi* within *Compsemydidae* will have to be confirmed in a broader context. Although this hypothesis strongly contrasts with previous concepts of paracryptodiran evolution, the independent loss of the palatine artery actually occurs relatively frequently in some turtle clades, such as in testudinoids, in which interspecific variation is important (Rollot et al., 2021). Further studies of paracryptodiran cranial anatomy should yield additional insights into the evolution of the basicranium and circulation system of paracryptodires.

### The articular surface of the occipital condyle

The exoccipitals are not involved in the formation of the functional articular surface of the occipital condyle in *Uluops uluops*, *Dorsetochelys typocardium* (pers. comm. of Jérémy Anquetin regarding the holotype), *Pleurosternon moncayensis* (Perez-Garcia et al., 2021), and, very likely, *Glyptops ornatus* (Gaffney, 1979a). The condition is unknown for *Compsemys victa* and *Pleurosternon bullockii* as the exoccipitals are either missing or badly preserved. The basioccipital and exoccipitals are fused in *Helochelydra nopcsai* (Joyce et al., 2011) and *Naomichelys speciosa* (Joyce et al., 2014) so that the contribution of the exoccipitals to the functional articular surface of the occipital condyle is unclear. The available skulls of *Aragochersis lignitesta* are too heavily damaged to allow any clear observation of bony contributions to the occipital condyle (Perez-Garcia et al., 2020). The occipital condyle in *Kallokibotion bajazidi* is illustrated as being formed by both the exoccipitals and basioccipital (Perez-Garcia & Codrea, 2018) but later studies showed that the occipital condyle is actually formed by the basioccipital only (Martín-Jimenez et al. 2021; personal observation, SWE, 2020). Gaffney (1982) described baenids as having their occipital condyle made up of both the basioccipital and exoccipitals, which is confirmed by more recent observations for *Cedrobaena putorius* (Lyson & Joyce, 2009b), *Neurankylus torrejonensis* (Lyson et al., 2016), *Peckemys brinkman* (Lyson & Joyce, 2009b), and *Saxochelys gilberti* (Lyson et al., 2019). In *Eubaena cephalica*, the potential contribution of the exoccipital to the occipital condyle cannot be determined due to the fusion of the basioccipital with the exoccipitals (Rollot et al., 2018). As a character for the bony contributions to the occipital condyle was missing in previous paracryptodiran matrices, we added a character in our matrix for this feature (our character 103). With current state of knowledge, the articular surface of the occipital condyle therefore appears to be formed by the basioccipital in *Pleurosternidae* and *Compsemydidae* and by both the basioccipital and exoccipitals in the remaining *Baenidae*.

### Phylogenetic relationships

Our initial phylogenetic analysis under equal weighting resulted in 168 most parsimonious trees with 298 character–state transitions. The strict consensus tree we obtained from that set of trees displays polytomies at the base of pleurosternids and at the base of baenodds. The “pruned trees” function in TNT was used to identify problematic taxa. Three rogue taxa were identified, namely *Scabremys ornata*, *Cedrobaena putorius*, and *Peckemys brinkman*, which were removed from our sample to run a second analysis. The second phylogenetic analysis resulted in 24 most parsimonious trees with 279 character–state transitions. The resulting strict consensus tree is shown in Fig. 10A. In this topology, *Uluops uluops* is retrieved as a pleurosternid along a polytomy within *Riodevemys inumbragigas*, *Dorsetochelys typocardium*, *Glyptops ornatus*, *Dinochelys whitei*, a clade formed by *Toremys cassiopeia* and *Pleurosternon bullockii*, and helochelydrids. Our phylogenetic analyses under implied weighting with K values of 3, 6, 9, and 12 including all original taxa all resulted in a single most parsimonious tree with a best score of, respectively, 30.68333, 19.55433, 14.41164, and 11.42283. Our results under different concavity constants are all nearly identical and only differ in minor aspects. Within pleurosternids, the observed source of variation concerns the phylogenetic placement of *Dorsetochelys typocardium* and *Riodevemys inumbragigas*. *Dorsetochelys typocardium* is alternatively found in a more basal position than *Riodevemys inumbragigas* (*K* = 3) or as sister taxon to the latter (*K* = 6, 12), while *Riodevemys inumbragigas* is sometimes retrieved in a more basal position than *Dorsetochelys typocardium* (*K* = 9). Within baenids, variation occurs in the placement of *Arundelemys dardeni* and *Trinitichelys hiatti* just outside of baenodds. *Arundelemys dardeni* is found in a more basal position than *Trinitichelys hiatti* under concavity constants of 9 and 12, whereas *Trinitichelys hiatti* is retrieved in a more basal position under concavity constants of 3 and 6. The single most parsimonious trees obtained from every analysis that employs implied weighting are all fully resolved and *Uluops uluops* is constantly found as the most basal pleurosternid. As the four topologies are extremely similar, we choose to present a single of these only, in particular the one obtained under implied weighting with a *K* value of 6 (Fig. 10B), as the position of *Arundelemys dardeni* and *Trinitichelys hiatti* differs from that in the strict consensus tree obtained under equal weighting.

Although the resolution within pleurosternids in the equal weight strict consensus tree is lower than any result obtained under implied weigthing, our analyses systematically retrieved the same global relationships within *Paracryptodira*, which represent novel results. In particular, *Uluops uluops* and *Helochelydridae* are placed within *Pleurosternidae*, whereas *Compsemydidae* including *Kallokibotion bajazidi* are located within *Baenidae* (Fig. 10). As the phylogenetic hypothesis shown in Fig. 10B represents a fully resolved tree by contrast to the topology shown in Fig. 10A, we base our rationale for the following section on this topology (see Additional file 2 for additional content about the other phylogenetic analyses).

*Uluops uluops* is united with all other pleurosternids by the presence of a jugal that does not extend deeply ventrally (character 19, state 1; polymorphic in *Naomichelys speciosa*), a large supraoccipital exposure on skull roof between parietals (character 63, state 2; small exposure in *Pleurosternon bullockii* and *Naomichelys speciosa*), an anteriorly convex nasal–frontal suture (character 69, state 1; straight in *Helochelydra nopcsai*), and the presence of anterior tubercula basioccipitale on the parabasisphenoid (character 99, state 1). Among other similarities existing between pleurosternids are the presence of a basipterygoid process which is large in helochelydrids (character 98, state 0), reduced in other pleurosternids (character 98, state 1), but absent in baenids (character 98, state 2), and the exclusion of the exoccipitals from the articular surface of the occipital condyle (character 103, state 1; homoplastically shared with *Kallokibotion bajazidi*), which likely contrasts with baenids in which a contribution of the exoccipitals is present (character 103, state 0). Within *Pleurosternidae*, *Uluops uluops* is retrieved as the most basal representative of the clade and differs from other taxa by having a foramen palatinum posterius entirely formed by the palatine (character 26, state 1), a length between orbit and cheek emargination equal to the diameter of the orbit (character 60, state 1; homoplastically present in *Pleurosternon bullockii*), and a maximum combined width of the parietals greater than their length (character 65, state 1; homoplastically present in *Kallokibotion bajazidi* and some baenids). The other members of *Pleurosternidae* are united by the presence of an elongate skull shape with a length that is more than twice the width (character 1, state 1; wedge-shaped skull in *Uluops uluops*), absence of lingual ridges (character 6, state 2; homoplastically shared with *Palatobaena* spp., anteriorly present in *Uluops uluops*), and a reduced to absent jugal contribution to the orbit margin (character 18, states 1 and 2; large contribution in *Uluops uluops*). The result and phylogenetic placement of *Uluops uluops* thus confirm the conclusion of Evers et al. (2020) that *Uluops uluops* is a pleurosternid. The overall shape of the skull, in particular the wedged-shape skull, therefore appears to be convergent upon that of baenids.

*Helochelydridae* is a clade of European and North American turtles known from Late Jurassic to Late Cretaceous deposits (Joyce, 2017). The monophyly of the group, as found in our analysis, appears to be robust as those turtles exhibit a unique skull, shell, and osteoderm surface texture consisting of distinct tubercles (Lapparent and Murelaga 1996; Joyce et al., 2011, 2014; Perez-Garcia et al., 2020). Although most of the material known is fragmentary, nearly complete shell and skull material is available for *Helochelydra nopcsai* (Joyce et al., 2011), *Naomichelys speciosa* (Joyce et al., 2014), and *Aragochersis lignitesta* (Perez-Garcia et al., 2020), which allowed the inclusion of those taxa in our analysis and comparison with pleurosternids. The inclusion of helochelydrids in an analysis of paracryptodiran relationships is, to our knowledge, the first of its kind. Although the monophyly of *Helochelydridae* is reliable, its relationship with other turtles remains unclear. Among others, *Naomichelys speciosa* was found as the sister to *Kallokibotion bajadizi* within the stem lineage of crown Cryptodira (Hirayama et al., 2000), as a stem turtle within *Meiolaniformes* (Anquetin, 2012), or as an independent lineage of stem turtles (Joyce et al., 2016; Sterli & de la Fuente, 2019). However, explicit affinities with pleurosternids had previously been suggested by Nopcsa (1928), who placed helochelydrids within his *Pleurosternidae*, and Evers et al. (2020), who identified similarities with *Pleurosternon bullockii*, such as the presence of anterior tubercula basioccipitale on the parabasisphenoid. The placement of helochelydrids within pleurosternids in our analysis represents a new phylogenetic hypothesis that appears to confirm the two aforementioned suppositions. *Helochelydridae* are placed within pleurosternids by the presence of a jugal that does not extend deeply ventrally (character 19, state 1; polymorphic in *Naomichelys speciosa*), a large supraoccipital exposure on skull roof between parietals (character 63, state 2; small exposure in *Pleurosternon bullockii* and *Naomichelys speciosa*), an anteriorly convex nasal–frontal suture (character 69, state 1; straight in *Helochelydra nopcsai*), and the presence of anterior tubercula basioccipitale on the parabasisphenoid (character 99, state 1). Helochelydrids are further united with all other pleurosternids but *Uluops uluops* by an elongate skull shape in dorsal view with a length that is more than twice the width (character 1, state 1), absence of a lingual ridge (character 6, state 2), and a reduced jugal contribution to the orbit margin (character 18, states 1 and 2). Helochelydrids are further united with the North American *Glyptops ornatus* and *Dinochelys whitei* based on the presence of a pointed snout shape (character 73, state 1; homoplastically present in some baenids). The three helochelydrid taxa included in our analysis form a monophyletic clade united by the presence of a large contribution of the frontals to the orbit margin (character 16, state 2), the absence of epiplastral processes (character 47, state 1; homoplastically shared with baenids), the absence of a labial ridge on the mandible (character 72, state 1; homoplastically shared with some baenids), the presence of procoelous or opisthocoelous cervical vertebrae (character 78, state 1; homoplastically shared with some baenids), the presence of raised tubercles on the shell (character 80, state 2), the presence of three suprapygals (character 88, state 2), the absence of contact between peripheral I and costal I (character 92, state 0; homoplastically shared with compsemydids), the presence of an entoplastral scute (character 104, state 1), and the presence of V-shaped anterior peripherals (character 105, state 1). We also note the symplesiomorphically shared presence between *Pleurosternidae*, *Proganochelys quenstedtii*, and *Kayentachelys aprix* of a large exposure of the prefrontal on the skull roof (as in *Compsemydidae* including *Kallokibotion bajazidi*), the presence of an ossified epipterygoid (as in *Compsemydidae* including *Kallokibotion bajazidi*), and a foramen distalis nervi vidiani exposed on the ventral surface of the skull (also present in some baenids). Although our matrix is generally used to investigate paracryptodiran in-group relationships, the placement of helochelydrids within pleurosternids highlights that close relationships between helochelydrids and historically termed pleurosternid turtles have to be considered in future studies. Redescriptions of other Late Jurassic to Early Cretaceous paracryptodiran taxa such as *Dorsetochelys typocardium*, *Glyptops ornatus*, or *Trinitichelys hiatti*, ideally using µCT scans, are expected to further clarify these relationships.

Another unusual finding of our analysis is the inclusion of *Kallokibotion bajazidi* within *Compsemydidae* and the placement of *Compsemydidae* within *Baenidae*, which contrasts with previous hypotheses (Joyce & Rollot, 2020; Lyson & Joyce, 2011; Perez-Garcia, 2012; Perez-Garcia et al., 2015a). *Compsemydidae* has remained an enigmatic group of turtles for more than a century. Possible affinities between *Compsemys victa*, the historically only recognized compsemydid taxon, and baenids (Gaffney, 1972a) or pleurosternids (Hutchison & Holroyd, 2003) had been suggested, but its phylogenetic relationships were only assessed for the first time by Lyson and Joyce (2011), who retrieved it as a stem baenoid. *Compsemys russelli* was then found as sister taxon to *Compsemys victa* (Perez-Garcia, 2012) and the name *Compsemydidae* was attributed to the clade formed by these two taxa shortly after (Perez-Garcia et al., 2015b). Subsequent studies confirmed the phylogenetic placement of *Compsemydidae* within *Paracryptodira* and found them just outside of Baenoidea (Joyce & Rollot, 2020; Perez-Garcia et al., 2015b). The Late Jurassic *Riodevemys inumbragigas* and *Selenemys lusitanica* were first retrieved as pleurosternids (Perez-Garcia et al., 2015a, 2015b) but placement within compsemydids was proposed as well (Joyce & Rollot, 2020). The Early Cretaceous *Peltochelys duchastelii* was successively thought to have close relationships with *Pleurodira* (Dollo, 1884), "Dermatemydidae" (Nopcsa, 1928), *Adocidae* (Chkhikvadze, 1975), *Nanhsiungchelyidae* (Nessov, 1977), and *Trionychia* (Meylan, 1988), but a recent reinterpretation of its lectotype suggested placement with *Compsemydidae* as well (Joyce & Rollot, 2020). Our results broadly conform to that of Joyce and Rollot (2020) in terms of compsemydid in-group relationships, with the exception that we included *Kallokibotion bajazidi* in our analysis and retrieved it as the most basal compsemydid, and that we do not find *Riodevemys inumbragigas* as a compsemydid but rather as a pleurosternid. Our analysis, however, found compsemydids within baenids (following phylogenetic definition of baenids by Lyson & Joyce, 2011) and not as sister group to baenoids. *Baenidae* is herein supported by the formation of the foramen praepalatinum by the premaxilla, maxilla, and vomer (character 24, state 1; foramen praepalatinum only formed by the premaxilla in some eubaenines), the presence of an extensive pterygoid-basioccipital contact owing to an elongate posterior process of the pterygoid (character 28, state 2), the absence of epiplastral processes (character 47, state 1; homoplastically shared with helochelydrids), well-developed axillary buttresses that broadly extend onto the costals (character 86, state 1), well-developed inguinal buttresses that clearly reach the costals (character 87, state 1), and the absence of basipterygoid processes (character 98, state 2). The monophyly of *Compsemydidae*, including *Kallokibotion bajazidi*, is based on a posterodorsal extension of the quadratojugal that does not extend over the cavum tympani (character 20, state 1), a midline contact of the pterygoids along 40–70% of their length (character 27, state 2; homoplastically shared with some palatobaenines and *Dorsetochelys typocardium*), the absence of cervical scutes (character 40, state 0; homoplastically shared with some pleurosternids), marginal I that is mainly located over peripheral I (character 46, state 2), a pointed snout (character 73, state 1; homoplastically shared with some pleurosternids and baenids), the absence of a contribution of the nuchal to the anterior shell margin (character 82, state 1), the absence of a contact between peripheral I and costal I (character 92, state 0; also in helochelydrids), and a superficial enclosure of the cavum tympani by a squamosal–quadrate contact (character 107, state 1).

In addition to finding compsemydids within baenids, another interesting result is the inclusion of *Kallokibotion bajazidi* within compsemydids. This hypothesis is partially based on our identification of several features in the anterior region of the shell, for which we explain our rationale below. Although we are aware that the relationships between *Kallokibotion bajazidi* and *Compsemydidae* need to be tested in a global context, these taxa exhibit a unique morphology of the nuchal region of the shell that is, to our knowledge, not found in any other turtle. One of the features found as a synapomorphy for this clade, the absence of a contribution of the nuchal to the anterior shell margin, is still under debate and varying hypotheses exist regarding the identity of the bony plates that form the anteriormost margin of the shell in *Kallokibotion bajazidi*. In most turtles, the nuchal plate forms the anteriormost part of the shell margin and is laterally bordered by the first pair of peripherals. In a handful of turtles, the nuchal is prevented from contributing to the anterior shell margin by a medial contact between peripherals I anterior to the nuchal. This condition is known with confidence in *Compsemys victa* (Gilmore, 1919), *Selenemys lusitanica* (Perez-Garcia & Ortega, 2011), *Compsemys russelli* (Perez-Garcia, 2012), and is thought to be present in *Peltochelys duchastelii* as well (Joyce & Rollot, 2020). These taxa are thus scored as such in our matrix. In *Kallokibotion bajazidi*, the bony plate that forms the anteriormost margin of the shell is a small rectangular plate of bone that contacts a pair of peripherals laterally and a polygony element posteriorly. Nopcsa (1923) identified the anterior element as a peripheral, Gaffney and Meylan (1992) as a nuchal, and Rabi et al. (2013a) and Perez-Garcia and Codrea (2018) as a subdivided nuchal (subdivided neural of Rabi et al., 2013a, which is likely a typographic error), but none provided an explicit rationale. We suggest that this anteriormost element corresponds to a supernumerary peripheral (or a fused pair of supernumerary peripherals). First of all, the attribution of this bony plate as being a nuchal seems initially implausible as no turtle is known to have serial nuchals. In part this is based on an embryological constraint, as the nuchal originates from a single pair of intermembranous precursors, likely the cleithra (Lyson et al., 2013; Smith-Paredes et al., 2021). In a number of turtles, namely *Compsemys russelli*, *Compsemys victa*, *Peltochelys duchastelii*, and *Selenemys lusitanica*, the elements that form the anteriormost margin of the shell and that are located anterior to the nuchal plate always correspond to the first pair of peripherals. Furthermore, a reanalysis of specimens referred to *Compsemys victa* (USNM 8549) suggests the presence of a supernumerary pair of peripherals in the anterior region of the shell, as four pairs, not three pairs, of peripheral elements are associated with the first costals. As we found these turtles to be the closest relatives of *Kallokibotion bajazidi* in our analysis, it is more parsimonious to interpret the rectangular bony plate anterior to the regular nuchal in *Kallokibotion bajazidi* as a peripheral element. To avoid circularity, we performed an additional analysis that omits all characters pertaining to the unusual homology of elements found in the nuchal area of compsemydids (characters 46, 82, 92, and 106 in our matrix). This modified analysis resulted in a single most parsimonious tree with nearly the exact same topology as the one presented herein, including strong support for the clade *Compsemydidae* to the inclusion of *Kallokibotion bajazidi*. The placement of *Kallokibotion bajazidi* within *Compsemydidae* and the interpretation of the marginal element of the shell as a peripheral therefore appear to be robust for the moment.

## Conclusion

We here describe the skull of the Late Jurassic (Tithonian) paracryptodire *Uluops uluops* based on µCT scans. Our results include a new phylogenetic hypothesis of paracryptodiran relationships and new insights into the carotid arterial circulation system of paracryptodires and its evolution. Our phylogenetic analysis of paracryptodiran relationships included for the first time *Helochelydridae* and *Kallokibotion bajazidi*, and retrieved helochelydrids within *Pleurosternidae*, and *Compsemydidae* including *Kallokibotion bajazidi* within *Baenidae*. The inclusion of *Kallokibotion bajazidi* within compsemydids is notably based on the retraction of the nuchal from the anterior margin of the shell, a unique feature only known in these taxa, for which we provide evidence herein. The placement of helochelydrids, a clade generally found along the turtle stem, within pleurosternids, suggests that close relationships between helochelydrids and historically termed pleurosternid turtles have to be considered in future works. We also demonstrate that the canalis caroticus lateralis for the palatine artery appears to be lost in most baenids and that its presence can only be confirmed with confidence in the basal pleurosternid *Uluops uluops* and the basal compsemydid *Kallokibotion bajazidi*, suggesting at least two independent losses of this structure within paracryptodires. Further studies on the internal cranial anatomy of paracryptodires are expected to improve our comprehension of the circulation system in those turtles.

## Supplementary Information

**Additional file 1**. Character-taxon matrix.

**Additional file 2**. Supplementary information file.

## Data Availability

The original set of µCT scans were deposited at the University of Colorado Museum of Natural History, Boulder, CO, USA, and will be made available to qualified researchers. 3D models of bones of UCM 53971 are available at Morphobank (http://morphobank.org/permalink/?P3919). The expanded matrix of Joyce and Rollot (2020) and additional information about characters used in this study are available in the Additional files 1, 2.
