# Supplementary material for: A redescription of the Late Jurassic (Tithonian) turtle Uluops uluops and a new phylogenetic hypothesis of Paracryptodira
Source: Swiss J Palaeontol. 2021 Oct 6;140(1):23. doi: 10.1186/s13358-021-00234-y (PMC8550081; doi:10.1186/s13358-021-00234-y)
Supplement: Supplementary file 2 — Additional file 2. Supplementary information file. [file 13358_2021_234_MOESM2_ESM.docx]

**Supplementary Information File**

**A redescription of the Late Jurassic turtle *Uluops uluops* and a new phylogenetic hypothesis of Paracryptodira**

Yann Rollot^1^, Serjoscha W. Evers^1^, Walter G. Joyce^1^

^1^ Department of Geosciences, University of Fribourg, Fribourg, Switzerland

**Characters modified from the matrix of Joyce & Rollot (2020)**

Character 16, frontal contribution to the margin of the orbit (lateral view): 0 = absent; 1= small; 2= large.

*Comment*: We found it difficult to rigorously distinguish between a large and small contribution of the frontal to the margin of the orbit within the sample of species in the matrix of Joyce & Rollot (2020) and had a hard time to identify the scorings. We have updated the matrix and meaning of characters states 1 = small and 2 = large with the inclusion of helochelydrids. Helochelydrids have an extremely large contribution of the frontal to the margin of the orbit in comparison to baenids, which are now scored as 1.

Character 19, ventral extension of the jugal: 0 = present, the jugal extends ventrally deep below the level of the orbit and may reach the ventral margin of the maxilla; 1 = absent, the jugal does not deeply extend ventrally and its ventral aspect does not extend below the level of the lower margin of the orbit.

Previous character definition: 0 = jugal extends ventrally as far as the maxilla; 1 = jugal ends ventrally before maxilla does.

*Comment*: We have changed the character definition and rescored taxa within our matrix. The jugal is now considered as extending ventrally as far as the maxilla only when it ventrally ends at the same level than the maxilla. *Arundelemys dardeni* changed from 1 to 0; *Peckemys brinkman* changed from 1 to 0; *Cedrobaena putorius* changed from 0 to 0/1; *Boremys pulchra* changed from 1 to “?”; *Palatobaena cohen* changed from 0 to 0&1.

Character 27 (former 28 in Joyce & Rollot, 2020), midline contact of pterygoid: 0 = pointed contact, less than 10% of total pterygoid-basisphenoid length; 1 = between 10% and 40% of total pterygoid-basisphenoid length; 2 = between 40% and 70% of total pterygoid-basisphenoid length; 3 = more than 70% of total pterygoid-basisphenoid length.

Previous character definition (Joyce & Rollot, 2020): Midline contact of pterygoid: 0 = large; 1 = small; 2 = absent.

*Comment*: This character is only applicable for taxa that lack the interpterygoid vacuity. Anterior limit for measurements is the pterygoid-vomer contact, posterior limit for measurements is the basisphenoid-basioccipital contact along the midline of the skull.

Character 28 (former 29 in Joyce & Rollot, 2020), presence of extent of pterygoid-basioccipital contact: 0 = absent; 1 = present; 2 = present and extensive.

*Comment*: We note that the pterygoid-basioccipital contact varies greatly in its extent among our ingroup. In the outgroups, this contact is absent. In a number of early paracryptodires, the two bones only form a short contact, mostly because the posterior process of the pterygoid ends about at the level of the basioccipital-pterygoid contact. In most baenids, by contrast, the posterior process of the pterygoid extends far behind the basioccipital-pterygoid contact and the pterygoid and basioccipital form an elongate contact. This character forms a morphocline and is therefore ordered herein.

Character 80 (former 86 in Joyce & Rollot, 2020), shell sculpturing: 0 = smooth to slightly sculptured; 1 = distinct tubercles for “*Glyptops*-like” sculpturing; 2 = raised tubercles that easily get knocked off.

*Comment*: A third state has been added to score helochelydrids within our matrix as they possess raised tubercles that easily get knocked off. Character is ordered.


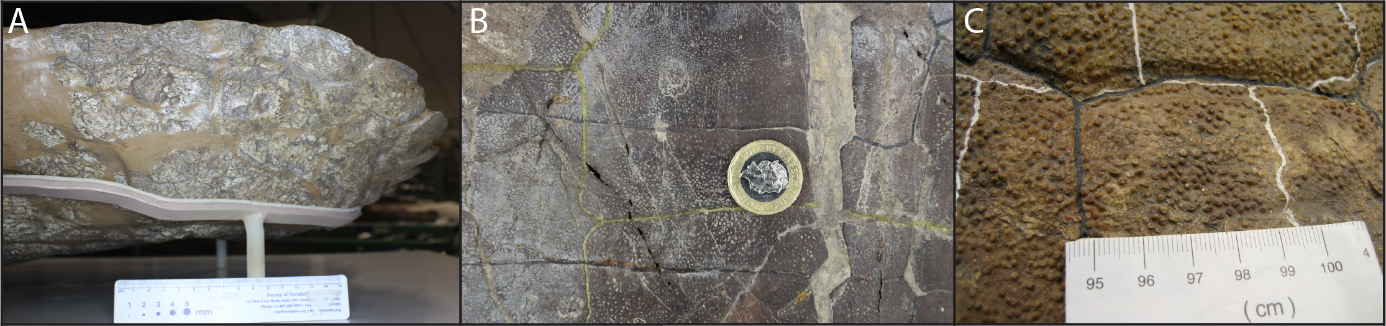


**Figure S1.** Illustration of character 80. **A**, state 0 (*Proganochelys quenstedtii*, SMNS 16980), **B**, state 1 (*Pleurosternon bullockii*, BMNH, 21351), and **C**, state 2 (*Naomichelys speciosa*, FMNH FR273).

Character 88 (former 94 in Joyce & Rollot, 2020), number of suprapygals: 0 = one; 1 = two; 2 = three.

*Comment*: A third state has been added to score *Naomichelys speciosa* and *Aragochersis lignitesta* (both helochelydrids) as they possess 3 suprapygals. Character is ordered.

**New characters added to the matrix of Joyce & Rollot (2020)**

Character 97, depth of upper temporal emargination relative to incisura columella auris in lateral view: 0 = absent to small, the deepest aspect of the upper temporal emargination does not extend further anteriorly than the deepest aspect of the incisura columella auris; 1 = intermediate, the deepest aspect of the upper temporal emargination located between the incisura columella auris and the anterior margin of the cavum tympani; 2 = deep, the deepest aspect of the upper temporal emargination extends further anteriorly than the anterior margin of the cavum tympani.

*Comment*: This character forms a morphocline that can be ordered.

Character 98, development of a basipterygoid process in ventral view: 0 = present and well-developed; 1 = present, but poorly developed; 2 = absent.

*Comment*: In the earliest known turtles, such as *Proganochelys quenstedtii* and *Kayentachelys aprix*, the basipterygoid processes are well-developed processes clearly visible in ventral view. In a number of early paracryptodires, the pterygoid is broadly sutured with the parabasisphenoid to immobilized the basiarticular joint, but remnants of the basipterygoid processes remain that are visible in ventral view. In late paracryptodires, these processes are generally absent. As we are unaware of any turtle that possesses basipterygoid processes that are not visible in ventral view, this character is specifically developed to refer to the visibility of these processes in ventral view, to allow scoring turtles for which CT scans are not available.

Character 99, anterior tubercula on the parabasisphenoid: 0 = absent; 1 = present.

*Comment*: The vast majority of turtles possess one pair of tubercula that are mostly formed by the basioccipitals and frame the basioccipital process. In some paracryptodires, but also helochelydrids, a second pair of tubercula is apparent further anteriorly that are mostly formed by the parabasisphenoid. In all cases, these tubercula are visible in the form of a raised process in ventral view.

Character 100, contribution of the prootic to the foramen nervi trigemini: 0 = present; 1 = absent, due to a contact of a posteroventral ramus of the parietal with the pterygoid along the posterior margin of the foramen nervi trigemini.

Character 101, enclosure of internal carotid artery in bone: 0 = internal carotid artery not enclosed in bone and foramen distalis nervi vidiani ventrally exposed; 1 = internal carotid artery canal anteriorly ossified but foramen distalis nervi vidiani ventrally exposed; 2 = internal carotid artery canal fully encased in bone and the foramen distalis nervi vidiani is not exposed ventrally.

*Comment*: This character pertains to the posterior portion of the internal carotid artery. We noticed differences in length of the internal carotid artery canal and variation of the placement and ventral exposure of several foramina related to the vidian nerve. The length of the internal carotid artery canal depends on the degree of ossification of the pterygoid posteriorly which also affects the coverage of various foramina. This supposes an ossification and enclosure in bone of the internal carotid artery towards the posterior, which is herein only considered valid for paracryptodires and not for other turtle clades such as plesiochelyids or sichuanchelyids that likely show a different ossification pattern of the basicranium.

In turtles that have character state 0, the internal carotid artery is not enclosed in bone, and the foramen distalis nervi vidiani (the distal foramina of the canalis pro ramo nervi vidiani) is exposed ventrally, generally among the posteriormost margin of the carotid pit/foramen posterius canalis carotici interni. Additionally, in these turtles, the foramen posterius canalis nervi vidiani is apparent along the anteriormost margin of the carotid pit/foramen posterius canalis carotici interni. This condition is typically illustrated by the circulation pattern of *Uluops uluops* (this paper). In turtles with character state 1, the internal carotid artery canal is anteriorly ossified but the foramen distalis nervi vidiani remains ventrally exposed, now more towards the anterior margin of the carotid pit/foramen posterius canalis carotici interni. The foramen posterius canalis nervi vidiani is not visible ventrally as it was in turtles with character state 0. The condition illustrated by character state 1 is typically present in a specimen of a new *Stygiochelys* species currently being described by Tyler R. Lyson (DMNS). In turtles with character state 2, the internal carotid artery is fully encased in bone posteriorly and the foramen distalis nervi vidiani is not exposed ventrally. The canalis pro ramo nervi vidiani thus joins the canalis caroticus internus instead of the ventral surface of the skull. This condition is typically illustrated by the circulation pattern of *Eubaena cephalica* (see Rollot et al. 2018).

Character 102, canalis caroticus lateralis: 0 = present; 1 = absent.

*Comment*: The vidian canal of some paracryptodires has previously been identified as the canalis caroticus lateralis. We here only score the canalis caroticus lateralis as absent, if descriptions in the literature explicitly outlines a morphology consistent with its absence and/or if we have access to CT scans.

Character 103, articular surface of the occipital condyle: (0) formed by the basioccipital and exoccipital; (1) formed by the basioccipital only.

*Comments*: state (1) in *Uluops uluops* that is shared with *Dorsetochelys typocardium* (pers. comm of Jérémy), *Glyptops ornatus*, and *Kallokibotion bajazidi*, unkown for *Compsemys victa* and *Pleurosternon bullockii*. Information in the literature is scarce, but Gaffney (1982) described baenids as having their occipital condyle made up of both the basioccipital and exoccipitals. A contribution of both bones to the occipital condyle has also been reported for *Cedrobaena putorius* (Lyson & Joyce, 2009), *Neurankylus torrejonensis* (Lyson et al., 2016), *Peckemys brinkman* (Lyson & Joyce, 2009), and *Saxochelys gilberti* (Lyson et al., 2019) but the extent of this contribution as well as if it reaches the articular surface of the occipital condyle is unclear. In *Eubaena cephalica*, the potential contribution of the exoccipital to the occipital condyle cannot be determined due to the fusion of the basioccipital with the exoccipitals (Rollot et al., 2018).

Character 104, entoplastral scute: 0 = absent; 1 = present.

*Comment*: We have added this character for *Aragochersis lignitesta*, *Helochelydra nopcsai*, and *Naomichelys speciosa* (all helochelydrids) as they possess an entoplastral scute, unlike other taxa in our sample.

Character 105, V-shaped anterior peripherals: 0 = absent; 1 = present.

*Comment*: We have added this character for *Aragochersis lignitesta*, *Helochelydra nopcsai*, and *Naomichelys speciosa* (all helochelydrids) as they possess V-shaped anterior peripherals, unlike other taxa in our sample.

Character 106, supernumerary peripheral elements: 0 = absent, peripheral ring consists only of 11 pairs of peripherals and a pygal; 1 = present, additional peripheral elements apparent.

*Comment*: We have added this character for *Kallokibotion bajazidi* and *Compsemys victa* as they possess a supernumerary peripheral element anterior to the nuchal, unlike other taxa in our sample.

Character 107, superficial enclosure of cavum tympani by squamosal/quadrate contact: 0 = absent; 1 = present.

*Comment*: We have added this character for *Kallokibotion bajazidi* and *Compsemys victa* as the cavum tympani of these taxa is superficially enclosed by a squamosal/quadrate contact, unlike other taxa in our sample.

**Characters deleted from the matrix of Joyce & Rollot (2020)**

Character 24, posterior extension of crista supraoccipitalis: 0 = extends beyond occipital condyle; 1 = does not extend beyond occipital condyle.

*Comment*: In comparison to more derived turtles where extremely prolonged crista supraoccipitalis, the difference among out sample are subtle and difficult to evaluate objectively, as most specimens are partial or crushed, because it is difficult to develop an objective reference point, and because we do not have access to appropriate photographs that would allow us to rescore many taxa. We therefore omitted/deleted this character entirely.

Character 31, basisphenoid size and shape: 0 = triangular; 1 = pentagonal; 2 = rectangular.

*Comment*: omitted/deleted because redundant with 28. The shape of the basisphenoid as meant with this definition is actually depending on the contact of the pterygoid with its counterpart.

Character 38, posterior margin of carapace: 0 = posterior margin rounded convex; 1 = posterior margin flat or concave.

*Comment*: omitted because redundant with character 55.

Character 69, internal carotid location: 0 = within basisphenoid; 1 = halfway between suture of pterygoid and basisphenoid.

*Comment*: omitted/deleted.

Character 78, lateral internal carotid: 0 = subequal to the internal carotid artery foramen; 1 = half the diameter of the internal carotid artery foramen.

*Comment*: We deleted this character, as the vast majority of paracryptodires, as far as known, lack the lateral canal entirely and because the canal is small in size whenever it is present. The character is therefore uninformative as phrased.

Character 84, fenestra perilymphatica: 0 = large; 1 = small.

*Comment*: The size of the fenestra perilymphatica has not actually been described for the vast majority of paracryptodires and for all taxa for which we have CT scans, it is not notably small. We therefore omit this character as irreproducible.

Character 98, distinct thickening on the lateral portion of the posterior plastral lobe: 0 = absent; 1 = present.

*Comment*: omitted herein as this character needs revision.

**Supplementary phylogenetic results**

The matrix used in this study is available as a separate file. The list of synapomorphies, the mapped synapomorphies, as well as the strict consensus and 50% majority-rule trees resulting from the analyses under implied weighting with K values of 3, 6, 9, and 12 are available as separate files at MorphoBank in the Documents section (http://morphobank.org/permalink/?P3919).
